# Human Cytomegalovirus Infection Upregulates the Mitochondrial Transcription and Translation Machineries

**DOI:** 10.1128/mBio.00029-16

**Published:** 2016-03-29

**Authors:** S. Karniely, M. P. Weekes, R. Antrobus, J. Rorbach, L. van Haute, Y. Umrania, D. L. Smith, R. J. Stanton, M. Minczuk, P. J. Lehner, J. H. Sinclair

**Affiliations:** a Department of Medicine, University of Cambridge Clinical School, Addenbrookes Hospital, Cambridge, United Kingdom; b Cambridge Institute for Medical Research, University of Cambridge, Cambridge, United Kingdom; c MRC, Mitochondrial Biology Unit, Cambridge, United Kingdom; d Paterson Institute for Cancer Research, University of Manchester, Withington, Manchester, United Kingdom; e Institute of Infection and Immunity, School of Medicine, Cardiff University, Cardiff, United Kingdom

## Abstract

Infection with human cytomegalovirus (HCMV) profoundly affects cellular metabolism. Like in tumor cells, HCMV infection increases glycolysis, and glucose carbon is shifted from the mitochondrial tricarboxylic acid cycle to the biosynthesis of fatty acids. However, unlike in many tumor cells, where aerobic glycolysis is accompanied by suppression of mitochondrial oxidative phosphorylation, HCMV induces mitochondrial biogenesis and respiration. Here, we affinity purified mitochondria and used quantitative mass spectrometry to determine how the mitochondrial proteome changes upon HCMV infection. We found that the mitochondrial transcription and translation systems are induced early during the viral replication cycle. Specifically, proteins involved in biogenesis of the mitochondrial ribosome were highly upregulated by HCMV infection. Inhibition of mitochondrial translation with chloramphenicol or knockdown of HCMV-induced ribosome biogenesis factor MRM3 abolished the HCMV-mediated increase in mitochondrially encoded proteins and significantly impaired viral growth under bioenergetically restricting conditions. Our findings demonstrate how HCMV manipulates mitochondrial biogenesis to support its replication.

## IMPORTANCE

Human cytomegalovirus (HCMV), a betaherpesvirus, is a leading cause of morbidity and mortality during congenital infection and among immunosuppressed individuals. HCMV infection significantly changes cellular metabolism. Akin to tumor cells, in HCMV-infected cells, glycolysis is increased and glucose carbon is shifted from the tricarboxylic acid cycle to fatty acid biosynthesis. However, unlike in tumor cells, HCMV induces mitochondrial biogenesis even under aerobic glycolysis. Here, we have affinity purified mitochondria and used quantitative mass spectrometry to determine how the mitochondrial proteome changes upon HCMV infection. We find that the mitochondrial transcription and translation systems are induced early during the viral replication cycle. Specifically, proteins involved in biogenesis of the mitochondrial ribosome were highly upregulated by HCMV infection. Inhibition of mitochondrial translation with chloramphenicol or knockdown of HCMV-induced ribosome biogenesis factor MRM3 abolished the HCMV-mediated increase in mitochondrially encoded proteins and significantly impaired viral growth. Our findings demonstrate how HCMV manipulates mitochondrial biogenesis to support its replication.

## INTRODUCTION

Human cytomegalovirus (HCMV) is a betaherpesvirus found in 50% to 90% of human populations worldwide. Infection of healthy individuals usually involves an asymptomatic acute phase followed by lifelong carriage in a latent form (1). However, HCMV is a serious pathogen under conditions of immunoincompetence, being a leading cause of morbidity and mortality during congenital infection, bone marrow or solid organ transplantations, or AIDS (1). HCMV drives major metabolic reprogramming of host cells during infection (2–5). Akin to tumor cells (6), HCMV infection leads to an increase in glucose uptake (7) by upregulation of glucose transporter 4 (Glut4) (8). During infection, the flux of carbon through glycolysis is increased (2, 3), and glucose-derived citrate is shuttled from the mitochondria to the cytosol (cataplerosis) for the biosynthesis of fatty acids, vital for viral envelopment (9). HCMV also drives an increase in glutamine conversion to α-ketoglutarate to fuel the tricarboxylic acid (TCA) cycle with carbon (anaplerosis) (10). While in some cancer cells and in budding yeast the increase in glycolysis under aerobic conditions is associated with suppression of mitochondrial respiration and oxidative phosphorylation (OXPHOS), known as the “Crabtree effect” (11, 12), HCMV infection is associated with an increase in both glycolysis (2) and mitochondrial respiration (13). Mitochondria play a central role in production of cellular energy and biosynthetic precursors and are key mediators and regulators of apoptosis and antiviral signaling (14). They contain autonomous genomes that are expressed by unique transcription and translation systems, yet the human mitochondrial genome encodes only 13 polypeptides (15). The vast majority of mitochondrial proteins (700 to 1,000 in humans), including all of the protein components of the mitochondrial transcription and translation machineries, are encoded by nuclear genes, are translated in the cytosol, and are imported into mitochondria using dedicated translocons (15).

HCMV infection is known to profoundly affect mitochondria and their function. Previous studies have indicated that mitochondrial DNA (mtDNA) synthesis is stimulated by HCMV infection (16) and that upregulation of OXPHOS genes occurs late during HCMV lytic cycle at the level of transcription (17), translation (18), and protein expression (19). An increase in endoplasmic reticulum-mitochondrial contact domains by HCMV infection has also been documented (20). While the HCMV antiapoptotic protein UL37x1 (21) causes fragmentation of the mitochondrial network (22), this is not associated with perturbed mitochondrial physiological functions and therefore is different from other pathological conditions associated with network disassembly (23). On the contrary, HCMV-infected cells are known to have increased labeling with the membrane potential-dependent dye tetraphenylphosphonium (TPP) (24) and show increased oxygen consumption, indicative of induction of respiration (13). Viral UL37x1 may be involved in this process since a UL37x1 knockout virus was partially impaired in induction of respiration (13); HCMV β2.7 long noncoding RNA has also been suggested to affect mitochondrial function by binding respiratory complex I (25). In this case, β2.7 RNA was required to sustain ATP production throughout viral infection (25).

How HCMV upregulates mitochondrial biogenesis and function is not well understood. Here, we have used quantitative mass spectrometry (MS) to measure the changes in the mitochondrial proteome following HCMV infection in order to understand these processes. We found that multiple proteins of the mitochondrial transcription and translation systems were induced early during the viral replication cycle. This was accompanied by an increase in the components of the respiratory chain complexes encoded by the mitochondrial genome. Proteins involved in mitoribosome biogenesis were markedly induced by HCMV infection. Knockdown of a recently identified member of this group, MRM3 (26, 27), abrogated virus induction of mitochondrially encoded proteins and significantly impaired viral growth under bioenergetically restricting conditions.

## RESULTS

### Mapping HCMV-induced changes in the mitochondrial proteome using SILAC-MS.

**FIG 1:**
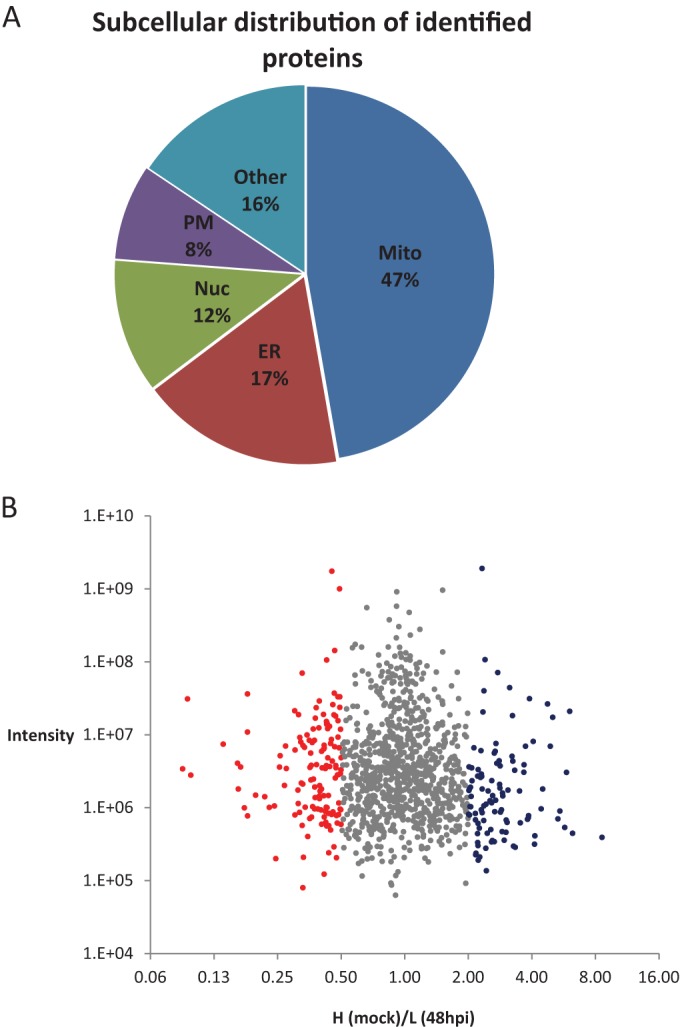
SILAC-MS analysis of the changes in the mitochondrial proteome following HCMV infection. (A) Subcellular localization annotation of 1,171 human proteins identified in isolated mitochondria with at least 2 unique peptides represented as a percentage of the total. Gene Ontology (GO) information was imported using the UniProt database. (B) Scatter plot representing fold change in “heavy” (mock)/“light” (HCMV 48 hpi) protein abundance in isolated mitochondria. A total of 12.3% (144) of proteins were increased over 2-fold following HCMV infection (red), 8.6% (101) were downregulated (blue), and 79.1% (926) were not significantly changed (gray).

To better understand how HCMV stimulates mitochondrial biogenesis and functions, we mapped the HCMV-induced changes in the abundance of mitochondrial proteins using quantitative mass spectrometry (MS) based on stable isotope labeling by amino acids in cell culture (SILAC). We carried out a SILAC-based screen in U373 astrocytoma cells, which in our previous studies appear to be more sensitive to changes in mitochondrial function mediated by HCMV than fibroblasts (25) and gave higher yields and purity of mitochondria (unpublished results). Three parallel cultures of U373 cells were differentially labeled with SILAC media (containing “light,” “heavy,” or “medium” amino acids) and then infected with the HCMV Merlin strain for 48 or 60 h or mock infected (see Fig. S1 in the supplemental material). Following infection, the three populations of cells were combined and mitochondria were isolated from the pooled-cell mixture by affinity purification. Lysed mitochondria were digested into peptides followed by MS. We identified 1,171 cellular proteins with at least 2 unique peptides in our mitochondrial preparation (see Table S1A in the supplemental material). According to Gene Ontology (GO) annotation, 47% of identified proteins were mitochondrial, while 17% and 12% were annotated to the closely associated organelles the endoplasmic reticulum (ER) and the nucleus, respectively (Fig. 1A). At 48 h postinfection (hpi), over 20% of identified proteins exhibited a specific and significant change using a 2-fold cutoff (Fig. 1B). Virus-induced changes to mitochondrial proteins persisted up to 60 hpi (see Fig. S2A in the supplemental material) and were reproducible (see Fig. S2B and Table S1B).

### HCMV reduces the association of mitochondria with proteins of other cellular structures and downregulates antiviral proteins.

To find functional groups within the proteins whose abundance was significantly changed by HCMV infection, we used the Database for Annotation, Visualization and Integrated Discovery (DAVID) (28). GO biological processes enriched within downregulated proteins included the categories “Cell adhesion,” “Actin cytoskeleton organization,” “Glycolysis,” and “Response to virus” (see Table S2A in the supplemental material). Reduction in actin binding proteins is consistent with HCMV disruption of the actin cytoskeleton (29–31). Given the documented activation of glycolysis by HCMV (2, 3), the observed reduction in glycolytic enzymes may appear counterintuitive. However, we found that glycolytic enzymes were not downregulated during infection in total cell extracts (our unpublished data); rather, the association of these enzymes with mitochondria was reduced in infected cells. Glycolytic enzymes are associated with mitochondria in mammalian cells (32). In plant cells (33, 34), the association of glycolytic enzymes with mitochondria is dynamically changed in response to respiratory demand (35). Whether HCMV-induced disassociation of glycolytic proteins from mitochondria has a metabolic role in HCMV replication remains to be determined. Of note, nonmitochondrial proteins were less associated with mitochondria upon infection, likely due to virus-induced fragmentation of the mitochondrial network (22).

In U373 cells, we observed a downregulation of several antiviral proteins (see Table S2A in the supplemental material). Most significant was an 8.3-fold reduction in the proapoptotic and proautophagic protein BCL-2/adenovirus E1B interacting protein 3 (BNIP3). We also observed viral induction of acetyl-coenzyme A acyltransferase 2 (ACAA2), which was shown to mitigate apoptosis induced by BNIP3 (36). A reduction of the antiviral signaling proteins MAVS and STING, which act in interferon beta (IFN-β) induction through the IFN regulatory factor 3 (IRF3) pathway, was also seen. This reduction is in line with HCMV-induced downregulation of IRF3 (19) and inhibition of IFN-β induction (37). A reduction in STING has also previously been observed in total cell extracts of HCMV-infected fibroblasts (19), although no changes in total amounts or subcellular localization of MAVS were observed (19, 38). MAVS may therefore undergo different fates in HCMV-infected fibroblasts and U373 cells. Our data point to a reduction in two additional viral restriction factors implicated in the interferon response: gamma-interferon-inducible protein 16 (IFI16) and phospholipid scramblase 1. IFI16 is required for activation of the IRF3 signaling cascade during human herpes simplex virus 1 (HSV1) infection (39). IFI16 was also shown to restrict HCMV replication, yet its inhibitory activity was not dependent on IFN-β induction (40). Phospholipid scramblase 1, a restriction factor of hepatitis B virus (41), was shown to augment the IFN response by increasing the expression of potent antiviral genes (42).

### Cellular pathways induced by HCMV infection.

DAVID analysis of HCMV-upregulated proteins showed enrichment of several cellular pathways, which included the KEGG_PATHWAY categories “Arginine and proline metabolism,” “Fatty acid metabolism,” and “One carbon pool by folate” (see Table S2B in the supplemental material).

Within arginine metabolism-related proteins, the highest induction (8.7-fold) was found in arginase II (ARG2). l-Arginine is the source for the synthesis of nitric oxide (NO), which plays a major role in host defense against microbial infection. In mouse macrophages, induction of arginase II was shown to prevent NO production by depleting intracellular arginine pools (43). Induction of arginase II was also previously detected during herpes simplex virus 1 (HSV-1) infection of cornea (44). Consequently, it may be that arginase II induction aids herpesviruses to suppress a host NO antiviral response.

Fatty acid synthesis is induced by HCMV infection (9). In particular, very-long-chain fatty acids are important for the production for infectious virions (45). HCMV-induced lipogenesis requires the inhibition of beta oxidation of long-chain fatty acids and is mediated by recruitment of the cellular protein viperin to mitochondria (46, 47). Interestingly, we found that proteins involved in beta oxidation of short and medium fatty acids were induced by HCMV (see Table S2B in the supplemental material). These included the mitochondrial short-chain-specific acyl coenzyme A (acyl-CoA) dehydrogenase (SCAD [2.3-fold induction]) and medium-chain-specific acyl-CoA dehydrogenase (MCAD [4.4-fold induction]), which catalyze the first step of oxidation of these fatty acids. It also included mitochondrial acetyl-coenzyme A acyltransferase 2 (ACAA2 [3-fold induction]), which catalyzes the last step of oxidation. In contrast, we found that the carnitine/acylcarnitine carrier protein, required for transport of long fatty acids into mitochondria, as well as the very-long-chain fatty acid-specific acyl-CoA dehydrogenase and trifunctional protein (TFP) complex, which catalyze beta oxidation of long-chain fatty acids, were not significantly changed by HCMV infection; this is consistent with the documented suppression of this pathway by HCMV (47). Whether a specific increase in the oxidation of short-chain fatty acids is supportive of HCMV replication remains to be determined. The induction of one-carbon metabolism in mitochondria is discussed below.

### Mitochondria from HCMV-infected cells were enriched with proteins containing predicted N-terminal mitochondrial targeting sequences.

By inspection of our list of mitochondrial proteins upregulated by infection, it appeared that mitochondrial matrix proteins are much more increased than proteins of the other mitochondrial subcompartments. As most matrix proteins contain N-terminal mitochondrial targeting peptides (mTP), we expected an enrichment of proteins displaying a high probability of containing an mTP in our data set. We identified mTP scores for all of our quantified proteins using the TargetP 1.1 server (48). We then plotted the TargetP score against the log heavy/light amino acid ratio (H/L ratio) (see Fig. S3A in the supplemental material). Proteins with a negative log ratio were relatively enriched in infected cells, which were labeled with light amino acids. We performed this analysis on data searched against the whole UniProt database (see Fig. S3Aa) and against the UniProt database of mitochondrial proteins only (annotated by Mitocarta [49]) (see Fig. S3Ab). In both cases, the averaged log (ratio) for high TargetP scores was significantly lower than the averaged log (ratio) for low scores, suggesting that proteins with high score of predicted mTP are indeed selectively enriched in HCMV-infected cells. Our findings may suggest that proteins containing an mTP are preferentially imported into mitochondria in infected cells. The reported increased accumulation of TPP in HCMV-infected cells (24) may indicate an increase in the mitochondrial membrane potential in infected cells, which is the driving force for import through the TIM complex into the matrix.

### HCMV specifically upregulated proteins involved in expression of the mitochondrial genome.

Thirty-three of the 144 (23%) proteins upregulated by HCMV function in expression and maintenance of the mitochondrial genome (Table 1). This group comprised ~40% of virally upregulated proteins annotated “mitochondrial” by GO, emphasizing the enrichment of this functional group among mitochondrial proteins upregulated by infection. This group included proteins associated with the mitochondrial nucleoid, proteins involved in mitochondrial mRNA transcription and turnover, mitochondrial tRNA maturation enzymes, mitochondrial tRNA synthetases, components of the small and large subunits of the mitoribosome, and proteins involved in mitoribosome assembly (Table 1). We have confirmed that the measured upregulation of mitochondrial expression proteins was not significantly biased by normalization due to different total amounts of heavy- and light-amino-acid-labeled mitochondrial proteins (see Fig. S3B and Text S1 in the supplemental material).

**TABLE 1:** Fold change and suggested functions of mitochondrial expression factors upregulated by HCMV infection

Protein	Gene(s)	H/L ratio (mock/48 hpi)	No. of unique peptides	Suggested mitochondrial function(s)	Reference(s)
mTERF domain-containing protein 1, mitochondrial	*MREF3*, *MTERFD1*	0.16	3	Mitochondrial ribosome biogenesis (mouse)	50
Mpv17-like protein 2	*MPV17L2*	0.19	2	Mitochondrial ribosome assembly (human)	51
Polymerase delta-interacting protein 2	*POLDIP2*	0.22	6	Mitochondrial nucleoid associated (human)	100
Putative methyltransferase NSUN4	*NSUN4*	0.25	3	Mitochondrial ribosome assembly (human, mouse)	52
28S ribosomal protein S27, mitochondrial	*MRPS27*	0.23	5	Small mitochondrial ribosome subunit (bovine)	101
GTPase Era, mitochondrial	*ERAL1*	0.25	7	Mitochondrial ribosome biogenesis (human)	53, 54
39S ribosomal protein L38, mitochondrial	*MRPL38*	0.26	7	Large mitochondrial ribosome subunit (bovine)	102
39S ribosomal protein L37, mitochondrial	*MRPL37*	0.27	8	Large mitochondrial ribosome subunit (bovine)	102
Dimethyladenosine transferase 2, mitochondrial	*TFB2M*	0.28	5	Mitochondrial transcription activation (mouse, human)	103
28S ribosomal protein S9, mitochondrial	*MRPS9*	0.28	12	Small mitochondrial ribosome subunit (bovine)	101
28S ribosomal protein S31, mitochondrial	*MRPS31*	0.30	4	Small mitochondrial ribosome subunit (bovine)	101
RNA methyltransferase-like protein 1	*MRM3*, *RNMTL1*	0.30	2	rRNA methyl transferase, mitochondrial ribosome biogenesis (mouse)	26, 27
GTP-binding protein 5	*GTPBP5*	0.31	3	Large mitochondrial ribosome associated, mitochondrial translation regulation (human)	104
Dimethyladenosine transferase 1, mitochondrial	*TFB1M*	0.34	4	Mitochondrial transcription (human)	103
ATP-dependent RNA helicase SUPV3L1, mitochondrial	*SUPV3L1*	0.35	9	DNA/RNA helicase, mitochondrial RNA turnover and processing (human)	105
Mitochondrial tRNA-specific 2-thiouridylase 1	*TRMU*	0.35	4	Mitochondrial translation regulation (human)	106
Probable asparagine-tRNA ligase, mitochondrial	*NARS2*	0.37	2	tRNA synthetase (human)	107
Mitochondrial ribonuclease P protein 1	*TRMT10C*, *MRPP1*	0.38	12	Mitochondrial tRNA maturation (human)	108
G-rich sequence factor 1	*GRSF1*	0.39	10	Mitochondrial mRNA turnover, mitochondrial ribosome biogenesis (human)	109
Putative ATP-dependent RNA helicase DHX30	*DHX30*	0.40	23	Mitochondrial translation regulation (human)	110
Tyrosine—tRNA ligase, mitochondrial	*YARS2*	0.42	6	Mitochondrial tRNA synthetase (human)	111
Methionyl-tRNA formyltransferase, mitochondrial	*MTFMT*	0.44	2	Mitochondrial translation (human)	112
28S ribosomal protein S29, mitochondrial	*DAP3*	0.44	11	Small mitochondrial ribosome subunit (bovine)	101
tRNA modification GTPase GTPBP3, mitochondrial	*GTPBP3*	0.44	4	Mitochondrial translation regulation (human)	113
SRA stem-loop-interacting RNA-binding protein, mitochondrial	*SLIRP*	0.45	3	Mitochondrial mRNA turnover (human)	114
Glycine—tRNA ligase	*GARS*	0.46	6	Mitochondrial tRNA synthetase (human)	115
28S ribosomal protein S22, mitochondrial	*MRPS22*	0.46	9	Small mitochondrial ribosome subunit (bovine)	101
Fast kinase domain-containing protein 5	*FASTKD5*	0.47	6	Mitochondrial translation (human)	110
Single-stranded DNA-binding protein, mitochondrial	*SSBP1*	0.48	8	mtDNA replication	116
Elongation factor G, mitochondrial	*GFM1*	0.49	13	Mitochondrial translation (human)	117
Fast kinase domain-containing protein 2	*FASTKD2*	0.49	9	Mitochondrial translation (human)	110
Zinc phosphodiesterase ELAC protein 2	*ELAC2*	0.49	9	Mitochondrial tRNA maturation (human)	118
28S ribosomal protein S17, mitochondrial	*MRPS17*	0.50	3	Small mitochondrial ribosome subunit (bovine)	101

The most significant upregulation within this group was in proteins only recently identified to play a role in mitoribosome biogenesis, a process which is still poorly understood. We observed a 6-fold viral induction of mTERF3 (50), a 5-fold induction of MPV17L2 (51) and a 4-fold increase in NSUN4 (52), ERAL1 (53, 54) and MRM3 (RNMTTL1) (26, 27). Translation of mitochondrial proteins, like bacterial proteins, requires an initiating formylated methionine. The formyl group is supplied by one-carbon metabolism (55). Consistent with this, we also observed an upregulation of enzymes that participate in one-carbon metabolism in the mitochondria, including mitochondrial bifunctional methylenetetrahydrofolate dehydrogenase (MTHFD2 [5.2-fold]), monofunctional C1-tetrahydrofolate synthase (MTHFD1L [2.2-fold]), and mitochondrial methionyl-tRNA formyltransferase (MTFMT [2.3-fold]).

Taken together, our SILAC data suggest that regulation of mitochondrial translation is a key process in viral induction of mitochondrial biogenesis. This is in line with a previous report showing that chloramphenicol (which specifically inhibits mitochondrial translation) abrogates HCMV induction of mitochondrial respiration (13).

In contrast to the upregulation of mitochondrial genome expression factors, the vast majority of nucleus-encoded proteins of the respiratory complexes were not affected by HCMV infection (see Table S3 in the supplemental material). Exceptions to this were several proteins suggested to be involved in assembly of the respiratory complexes, which involves the integration of nuclearly and mitochondrially encoded proteins (highlighted in green in Table S3). These proteins included the complex I assembly factors, evolutionarily conserved signaling intermediate in Toll pathway (ECSIT), Mimitin (NDUFAF2) and NDUFAF7, and complex III mitochondrial chaperone BCS1, as well as complex IV cytochrome *c* oxidase assembly factor 7.

### Upregulation of mitochondrial expression factors occurs early during the HCMV replication cycle.

**FIG 2:**
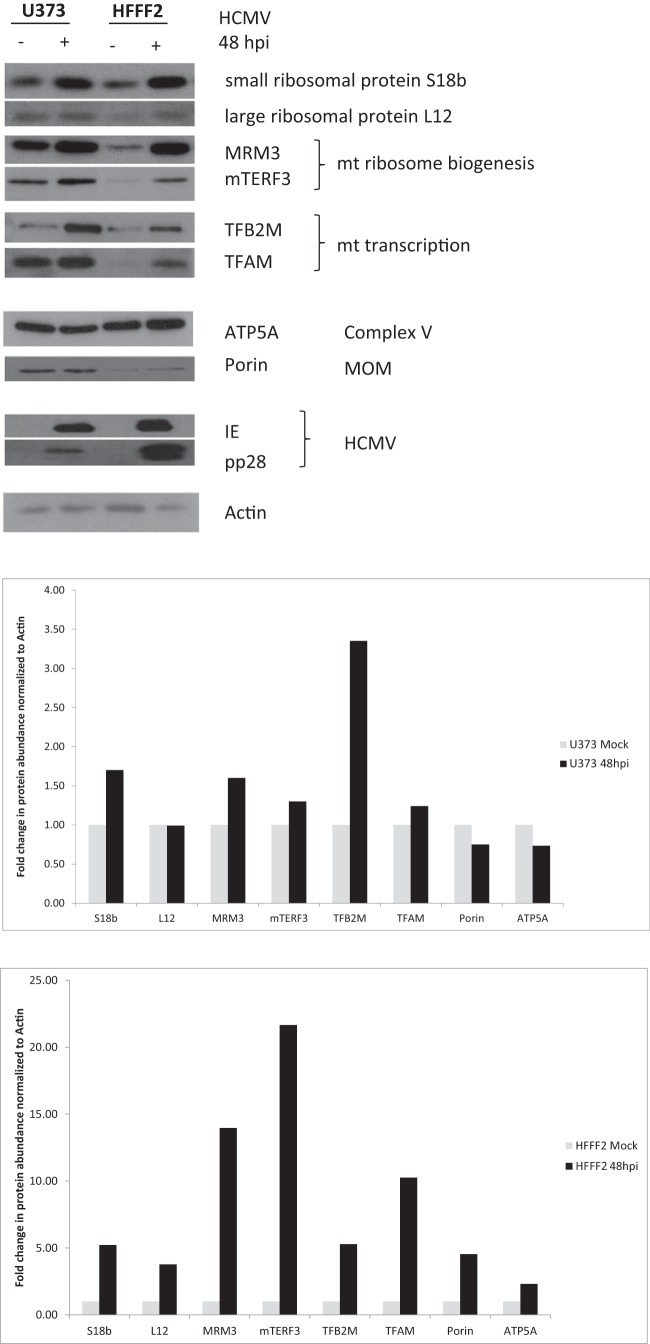
Upregulation of mitochondrial translation and transcription factors in HCMV-infected cells. U373 and human fibroblast (HFFF2) cells were either mock infected or infected with HCMV (Merlin) at an MOI of 5 for 48 h. WB analysis of total cell extracts using the indicated antibodies is shown. The fold change in protein abundance measured by WB is presented in the column charts.

We next used Western blot (WB) analysis to confirm the changes detected in mitochondrial expression factors in our SILAC-MS analysis and obtained a good correlation of measurements between methods (see Fig. S2C in the supplemental material). Consistent with our SILAC-MS data, we observed an upregulation of components of the small and large mitoribosomal subunits (MRPS 18b, with MRPL12 more significant for the former), mitochondrial ribosome biogenesis factors (mTERF3 and MRM3), and mitochondrial transcription factors (TFAM and TFB2M) in U373 cells at 48 hpi (Fig. 2, left lanes). ATP5A a nuclearly encoded subunit of respiratory complex V, and the mitochondrial outer membrane protein porin (VDAC1) were not upregulated by HCMV infection of U373 cells, consistent with our SILAC data. We also analyzed if this upregulation occurs during HCMV infection of human fibroblasts, where induction of OXPHOS genes was previously shown (17, 19), and found that upregulation of mitochondrial expression proteins was more pronounced in HCMV-infected fibroblasts (Fig. 2, right lanes). In line with our SILAC-MS data, a minor upregulation of ATP5A and porin was observed in infected fibroblasts, consistent with the documented overall increase in mitochondrial biogenesis in these cells (13, 17, 19).

**FIG 3:**
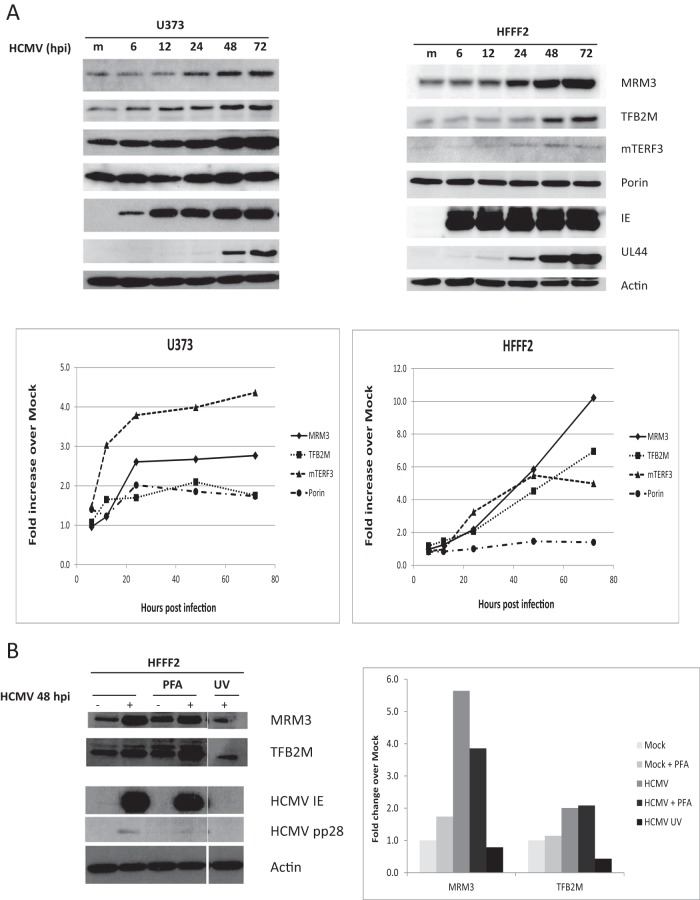
Upregulation of mitochondrial expression factors occurs during the early phase of HCMV replication. (A) Time course analysis. U373 and HFFF2 cells were either mock infected or infected with HCMV (Merlin) at an MOI of 5 for the times indicated. Lysates were processed and analyzed as described in the legend to Fig. 2. Membranes were labeled with the indicated antibodies. The upregulation of mitochondrial expression factors was first observed at 24 hpi concomitant with the expression of the HCMV UL44 early protein. m., mock infected cells. (B) Induction of mitochondrial expression factors is dependent on the expression of immediate early/early but not late HCMV genes. HFFF2 cells were either mock infected or infected with an intact or a UV-inactivated HCMV (Merlin) at an MOI of 5 for 48 h.

We performed a time course analysis to determine the kinetics of viral induction of mitochondrial expression factors using MRM3 and TFB2M as representative proteins of the mitochondrial translation and transcription machineries. In both U373 and fibroblast cells, the accumulation of MRM3 and TFB2M was evident by 24 hpi, and they continued to accumulate through the viral replication cycle (Fig. 3A). Since upregulation was more robust in fibroblasts, we continued our analysis in these cells. mtDNA copy number also increased during HCMV infection at later stages of the viral replication cycle, in agreement with previous reports (see Fig. S4 in the supplemental material) (13, 16). This increase could lead to an induction of mitochondrially encoded transcripts (mRNAs and rRNAs). However, an increase in the levels of mitochondrially encoded mRNAs *per se* may not be sufficient (or even required) for increased synthesis of mitochondrially encoded proteins as it was recently suggested that mammalian mitochondria (*in vivo*) contain a great excess of mitochondrial transcripts (56). Of note, the 2.7-fold increase in mtDNA observed at 72 hpi is comparable to the ~3-fold increase in mtDNA synthesis (as measured by ^3^H-dT incorporation) at 68 hpi observed by Furukawa et al. (16) and the 3.5-fold increase in mitochondrial mass (as assessed by nonyl acridine orange staining) observed by Kaarbø et al. (13). The ~300-fold increase in mtDNA at 72 hpi reported by Kaarbø et al. (13) was higher than our data suggest.

Our results suggest that the upregulation of MRM3 and TFB2M involves early or possibly immediate early (IE) viral gene expression. Consistent with this, no upregulation of MRM3 and TFB2M was observed when cells were infected with a UV-inactivated virus (which failed to produce the viral IE proteins) (Fig. 3B), and their induction was not perturbed by the viral DNA polymerase inhibitor phosphonoformic acid (PFA), which blocks viral late gene expression (marked by HCMV pp28) (Fig. 3B). The induction of MRM3 and TFB2M was also observed at the level of mRNA (see Fig. S5 in the supplemental material), supporting the view that viral regulation of these genes is likely to be transcriptional.

### HCMV genes known to target mitochondria are dispensable for induction of mitochondrial expression factors.

**FIG 4:**
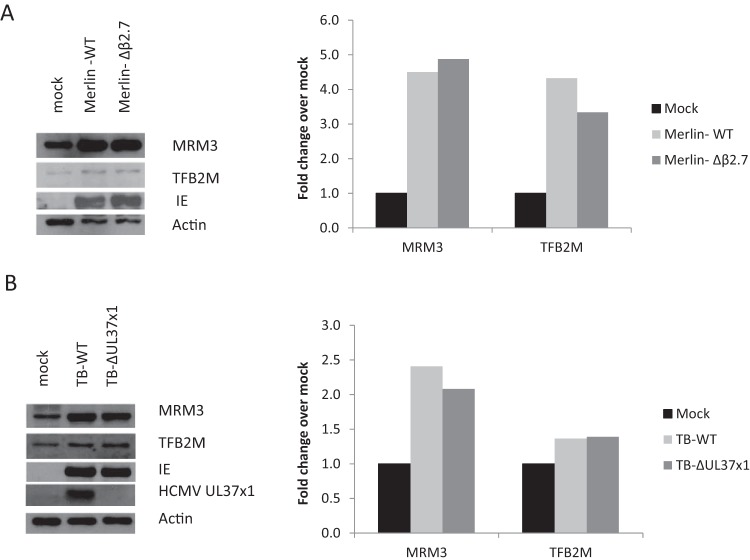
HCMV β2.7 and UL37x1 are not required for the induction of mitochondrial expression factors. HFFF2 cells were either mock infected or were infected for 48 h with wild-type (WT) HCMV (Merlin-BAC in panel A or Towne-BAC in panel B) or mutant viruses with the β2.7 (A) or UL37x1 (B) genes deleted.

Two HCMV gene products have previously been shown to affect mitochondrial respiration: the UL37x1 protein and the long noncoding β2.7 RNA. A UL37x1 knockout virus was partially impaired in induction of respiration (13), while a β2.7 knockout virus failed to maintain ATP production throughout the viral infection cycle (25). We therefore determined whether the UL37x1 and β2.7 RNA genes are important for the induction of mitochondrial expression factors observed at early stage of HCMV replication. We found that neither UL37x1 nor β2.7 is required for viral induction of MRM3 and TFB2M (Fig. 4). The identity of the viral gene or genes that govern this induction remains to be determined.

### HCMV induces the production of mitochondrially encoded proteins.

**FIG 5:**
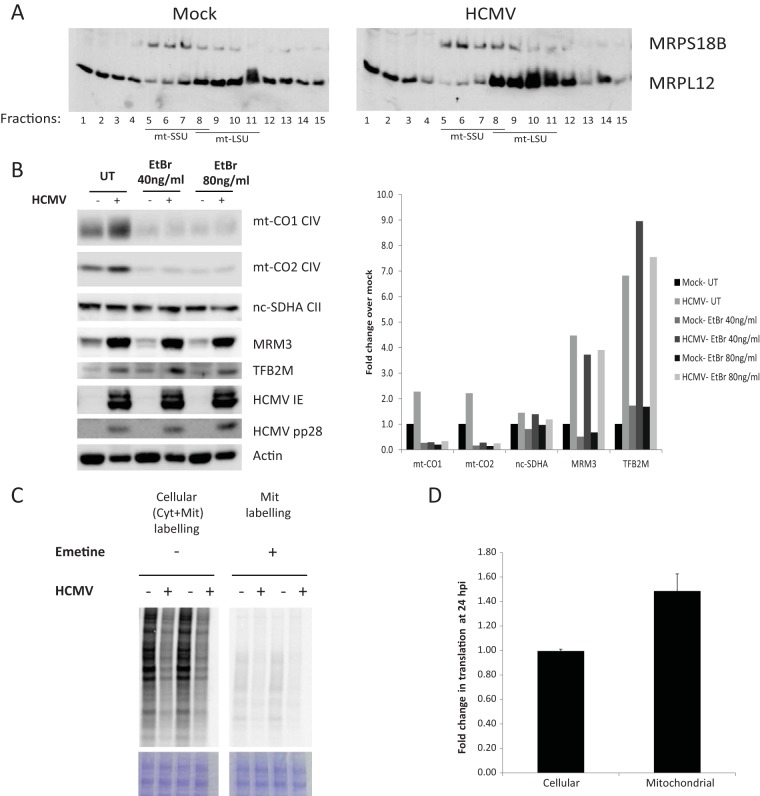
HCMV induces the synthesis and stabilization of mitochondrially encoded proteins. (A) Assembled mitoribosomes are induced by HCMV. Equal amounts of proteins of total cell lysates from mock-infected or HCMV-infected HFFF2 cells at 48 hpi were separated on a linear sucrose gradient (10 to 30% [wt/vol]) and analyzed by WB. Free nonassembled ribosomal subunits (SSU, small subunit; LSU, large subunit) migrate to the lower fractions, while assembled mitoribosomes appear in the higher-density fractions. (B) Induction of mitochondrially encoded proteins by HCMV. HFFF2 cells were either mock infected or were infected with HCMV at an MOI of 5. After 2 h, the inoculum was washed, and cells were refreshed with untreated medium (UT) or with medium containing ethidium bromide (EtBr) to block mitochondrial transcription. Cells were harvested at 48 hpi, and lysates were processed and analyzed as described in the legend to Fig. 2. The chart shows the fold change in protein abundance. HCMV induces mitochondrial translation. (C to D) Mock-infected and HCMV-infected cells were radiolabeled with [^35^S]methionine at 24 hpi in the presence of emetine (which blocks cytosolic translation) to determine mitochondrial translation or in the absence of emetine to determine cellular translation. Total cell lysates were separated on a 4 to 12% Bis-Tris Plus PAGE. Equal loading of the gels was confirmed by staining the gel with Coomassie brilliant blue G-250. Dried gels were exposed to a phosphorimager screen and visualized using a phosphorimager scanner (C). Radioactive counts in cell lysates were measured directly or after TCA precipitation and used to calculate cellular and mitochondrial translation efficiencies, as described in the text. The chart shows the fold change in translation after infection (D).

Having demonstrated that HCMV upregulates proteins of the mitochondrial transcription and translation machineries, we asked whether mitochondrial translation and the end products of these processes, mitochondrially encoded proteins, are also increased by HCMV infection. We first analyzed the assembly of mitochondrial ribosomal proteins following infection using sucrose gradient separation. We found an increase in assembled mitoribosomal subunits in the dense fractions using antibodies against the large (MRPL12) (Fig. 5A) and small (MRPS18b [Fig. 5A] and MRPS17 [not shown]) ribosomal subunits.

We next looked at steady-state levels of mitochondrially encoded proteins and found that mtDNA-encoded cytochrome oxidases 1 and 2 (mt-CO1 and mt-CO2), components of respiratory complex IV, were both upregulated after 48 h of infection of fibroblasts (Fig. 5B; see Fig. S6A in the supplemental material). In contrast, the levels of nuclearly encoded succinate dehydrogenase A (SDHA), part of respiratory complex II, were not significantly changed by infection. Of note, a moderate increase in mt-CO2 was observed in HCMV-infected U373 cells only at 72 hpi (see Fig. S6B) and not at 48 hpi (see Fig. S6B and Table S1A and S1B in the supplemental material). The increased upregulation of mitochondrially encoded proteins in infected fibroblasts compared to U373 cells correlates with the robust upregulation of mitochondrial transcription and translation factors in the former, as shown in Fig. 2.

We then followed mitochondrial translation by radiolabeling mock-infected and HCMV-infected cells with [^35^S]methionine in the presence of emetine, which specifically blocks cytosolic (but not mitochondrial) translation. When equal amounts of cell lysates were separated by SDS-PAGE and analyzed by autoradiography, we found that radiolabeling of mitochondrially encoded proteins was reduced after 24 h of HCMV infection (Fig. 5C, right lanes; see Fig. S6C, lanes 1 and 2, in the supplemental material). This was surprising since our WB analysis (Fig. 5B) suggested an increase in their steady-state levels. Moreover, we observed a significant decrease in the radiolabeling of cellular proteins (when labeling in the absence of emetine; Fig. 5C, left lanes). Since HCMV, unlike other viruses, maintains cellular translation and mTOR activation (57), we questioned if the decrease in radiolabeled proteins we observed resulted from a specific reduction in translation or reflected a reduction in [^35^S]Met uptake by infected cells under our experimental conditions. We, therefore, measured trichloroacetic acid (TCA)-precipitable counts ([^35^S]Met incorporated into proteins) and total radioactive count (including all [^35^S]Met taken up by the cells) in mock- and HCMV-infected cell lysates. We found that, indeed, total [^35^S]Met counts in infected cells (incorporated and nonincorporated [^35^S]Met) were reduced compared to those in mock-infected cells, suggesting that less [^35^S]Met was taken up by infected cells. We then calculated cellular and mitochondrial translation by dividing the TCA-precipitated counts by total counts, which represents the efficiency of [^35^S]Met incorporation into proteins and corrects for the biases in uptake of [^35^S]Met into the cells. When the above was taken into account, we found that mitochondrial translation is specifically induced at 24 h post-HCMV infection, while rates of cellular translation were similar between infected and uninfected cells (Fig. 5D). Nonetheless, we cannot exclude the possibility that HCMV perturbed cellular translation, which was accompanied by a decrease in uptake of [^35^S]Met, as translation inhibition was previously suggested to restrict uptake of at least some amino acids (58). In this case, our data would suggest a relative increase in mitochondrial translation over cytosolic translation in infected cells but not an absolute increase in mitochondrial translation compared to that in uninfected cells.

Interestingly, when uninfected cells were pretreated for 4 h with emetine prior to radiolabeling, mitochondrial translation was essentially blocked, while translation in HCMV-infected cells was maintained (see Fig. S6C in the supplemental material). It was previously shown that inhibition of cytosolic translation blocks mitochondrial translation within several hours, probably due to an elimination of a short-lived nucleus-encoded mitochondrial translation factor or factors (59). In sucrose gradients, we have observed an increase not only in assembled ribosomal subunits but also in nonassembled MRPL12 (Fig. 5A, fractions 1 to 4). Having a pool of mitochondrial translation factors (including nonassembled ribosomal subunits) may allow infected cells to retain mitochondrial translation under stress that blocks cytosolic translation.

To determine if the stability of mt-CO1 and mt-CO2 was also affected by viral infection, we blocked mitochondrial translation at 24 hpi using chloramphenicol and monitored the mt-CO1 and mt-CO2 levels at several time points. We found that the stability of both mt-CO1 and mt-CO2 was increased by infection (see Fig. S6D in the supplemental material). Blocking either mitochondrial transcription, using ethidium bromide (EtBr) (Fig. 5B), or mitochondrial translation (see Fig. S6A) immediately after viral inoculation caused, as expected, an almost complete elimination of mt-CO1 and mt-CO2 but not of nucleus-encoded SDHA from both infected and uninfected cells.

This inhibition of mitochondrial transcription and translation did not feed back to the nucleus to block expression of nucleus-encoded MRM3 and TFB2M (which are required for these processes); viral induction of both proteins was not impaired by EtBr or chloramphenicol treatments (Fig. 5B; see Fig. S6A in the supplemental material).

### Inhibition of mitochondrial ribosome biogenesis interferes with viral growth.

**FIG 6:**
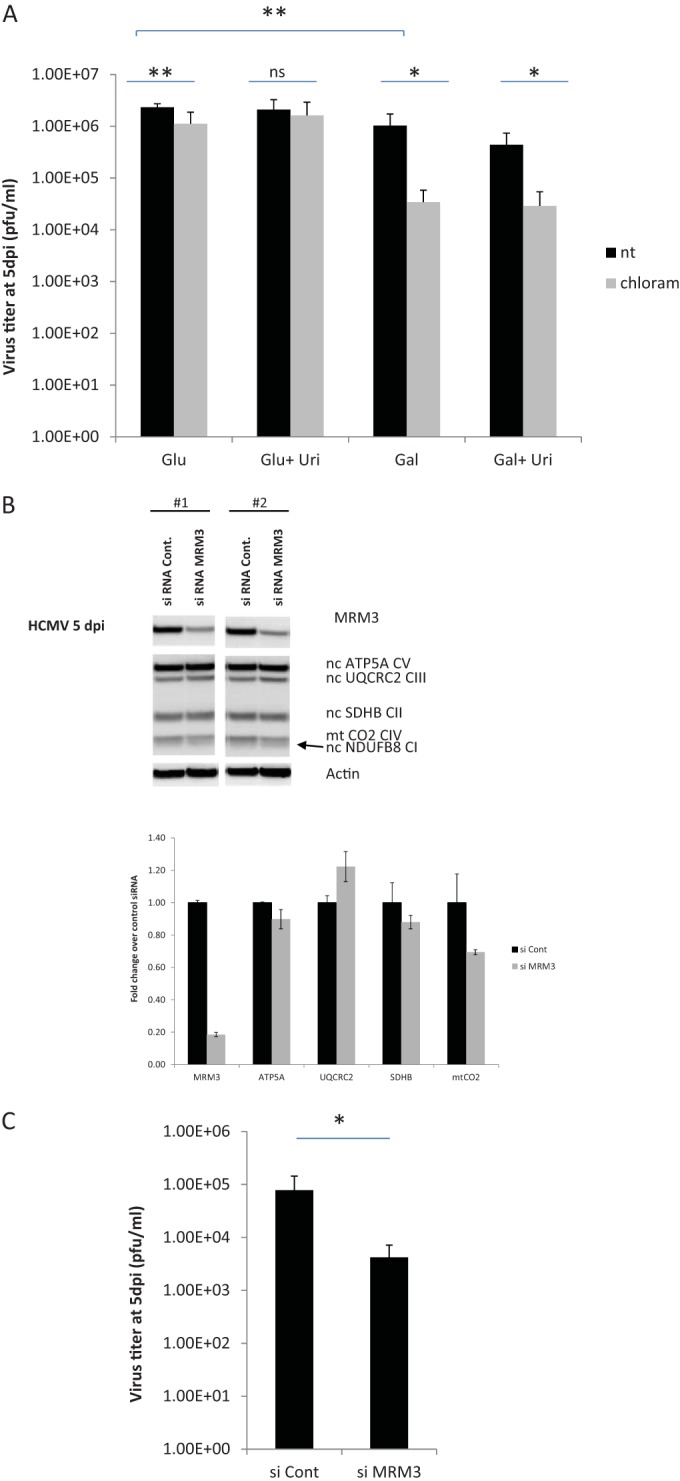
Inhibition of mitochondrial translation reduces virus titers. (A) HFFF2 cells were infected with HCMV at an MOI of 5 for 1 h and then washed and refreshed with DMEM supplemented with 10% dialyzed FBS, 2 mM glutamine, and antibiotics (DMEM-10dFBS) and 5 mM glucose. At 24 hpi, cells were washed and refreshed with DMEM-10dFBS containing either 5 mM glucose or 5 mM galactose with or without the addition of 0.2 mM uridine. Each medium was either left nontreated (nt) or was treated with chloramphenicol (50 µg/ml) to block mitochondrial translation. At 5 dpi, media were collected from cells, and released virus titers in supernatants were quantified using the 50% tissue culture infective dose (TCID_50_). Error bars represent the standard error of the mean (SEM) from two experiments with three replications each. (B and C) HFFF2 cells were transfected with a control siRNA or an siRNA targeting MRM3. After 22 h of transfection, cells were infected with HCMV (Merlin) at an MOI of 3, and at 24 hpi, cells were washed and refreshed with DMEM-10dFBS containing 5 mM galactose. At 5 dpi, media were collected from infected cells, and virus titers were quantified using the TCID_50_ (C). Cells were lysed and analyzed by WB; the results of two biological repeats are shown (B). The chart shows the fold change in protein abundance compared to control siRNA-treated cells. Error bars in panel C represent the standard errors from two biological repeats, each performed with three transfection replicates. *, *P* < 0.05; **, *P* < 0.001 (unpaired *t* test with Welch’s correction).

Why might HCMV require an increase in mitochondrial gene expression? Blocking mitochondrial transcription or translation after viral infection did not affect the accumulation of immediate early or late viral proteins at 48 hpi, suggesting that expression of the mitochondrial genome is not required for viral gene expression (Fig. 5B; see Fig. S6A in the supplemental material). To evaluate the importance of mitochondrial translation for viral growth, we blocked mitochondrial translation after 24 h of infection (the earliest time point where we observed an induction in mitochondrial translation factors) and measured released virus titers at 120 hpi. Chloramphenicol treatment caused a statistically significant, yet very minor, ~2-fold reduction in virus titers when cells were grown on Dulbecco’s modified Eagle’s medium (DMEM) supplemented with glucose (5 mM) (Fig. 6A, compare the 1st column pair), a similar effect was reported previously (13). While not leading to cell death (not shown), this mild effect may result from a slightly poorer health of chloramphenicol-treated cells. No significant effect on titers was seen earlier during infection (at 3 days postinfection [dpi]), yet treatment of cells with chloramphenicol 24 h prior to infection caused a >0.5-log reduction in viral titers (see Fig. S6E in the supplemental material). Importantly, we observed a robust >1.5 log reduction of viral growth when mitochondrial translation was blocked under more restrictive conditions. For instance, cell growth on galactose as a major carbon source is more dependent on oxidative phosphorylation and is thus routinely used to test respiratory competence of cells (60). When HCMV-infected cells were shifted to growth on galactose at 24 hpi, only a minor reduction in virus titers at 120 hpi was observed compared to growth on media containing glucose (Fig. 6A, compare black columns 1 and 3). However, a significant >1.5-log reduction in virus titers occurred when chloramphenicol was added to galactose-fed infected cells (Fig. 6A, compare the 3rd column pair), suggesting an important role for mitochondrial translation under demanding respiratory conditions. No significant death of infected cells was associated with growth on galactose either with or without chloramphenicol (data not shown). Impaired growth of cultured cells treated with chloramphenicol is driven by pyrimidine auxotrophy due to the requirement of a mitochondrial electron transport chain for the activity of dihydroorotate dehydrogenase (DHOdehase), a key enzyme in pyrimidine biosynthesis (61). Accordingly, supplementation of chloramphenicol-treated cells with uridine restores their growth to normal levels (61). Since pyrimidine biosynthesis is important for HCMV replication (62), and our SILAC-MS data show HCMV induction of DHOdehase (see Table S1A in the supplemental material), we asked if supplementation of HCMV-infected cells with uridine would augment viral growth when mitochondrial translation was inhibited. However, uridine addition alone could not alleviate the inhibition of viral production on cells grown on galactose in the presence of chloramphenicol (Fig. 6A, compare 4th column pair). Thus, the requirement of mitochondrial translation is not due to impairment of pyrimidine production. This fits our observation that chloramphenicol does not prevent late viral protein production (Fig. 5B), as would be expected if pyrimidine biosynthesis was impaired. Taken together, our findings suggest that chloramphenicol inhibits viral replication due to an energetic deficiency.

Our SILAC-MS data demonstrated that proteins involved in mitochondrial ribosome biogenesis are highly induced upon HCMV infection. On this basis, we asked if depletion of such a virally induced protein could prevent the induction of mitochondrial translation and impair viral growth. Consequently, we transfected fibroblasts with either a control small interfering RNA (siRNA) or siRNA directed against the virus-induced mitoribosomal biogenesis factor MRM3. After 16 h, cells were infected with HCMV and shifted to galactose medium at 24 hpi. At 120 hpi, we measured viral titers and analyzed cells by WB analysis. We found that depletion of MRM3 (~20% relative to control siRNA) abrogated viral induction of the mitochondrially encoded CO2 (mt-CO2). In contrast, nucleus-encoded OXPHOS proteins were not significantly affected by MRM3 depletion (Fig. 6B). Concomitantly, knockdown of MRM3 significantly reduced viral titers (Fig. 6C), which mimicked the effect of chloramphenicol (Fig. 6A). Thus, viral induction of this mitoribosomal assembly factor appears important for viral growth.

## DISCUSSION

HCMV has a profound effect on the expression of mitochondrial genes as well as on mitochondrial organization and functions. Here, we have affinity purified mitochondria and used quantitative MS to map the HCMV-mediated changes in the mitochondrial proteome. We found that in HCMV-infected U373 cells, over 20% of mitochondrially associated proteins were significantly modulated. We identified several antiviral factors that were downregulated by HCMV infection, which included proteins involved in regulation of the interferon response, a major target of HCMV (37). Interestingly, we found that a large group of mitochondrial proteins—all involved in mitochondrial gene expression—were significantly upregulated during infection. This upregulation was more robust in infected fibroblasts than in U373 cells, which is consistent with the more efficient viral replication in fibroblasts (63). The highest fold change occurred in proteins involved in the biogenesis of the mitoribosome. Although, the structure of the mitoribosome was recently resolved at high resolution (64), the process of mitoribosome biogenesis is still largely unknown, and most mitochondrial biogenesis factors we found to be virally induced have only recently been functionally annotated. It is tempting to speculate that additional mitochondrial proteins upregulated by HCMV infection that currently have no annotation might have a role in mitoribosome biogenesis.

HCMV induction of proteins involved in mitochondrial transcription and translation was correlated with an increase in [^35^S]methionine incorporation into mitochondrial proteins followed by an increase in steady-state levels of mitochondrially encoded proteins (Fig. 5). Interestingly, mitochondrially encoded proteins were induced by HCMV infection at times when gene expression of nucleus-encoded mitochondrial proteins was unchanged. Thus, the upregulation in the mitochondrial transcription and translation apparatuses may prime the biogenesis of the mitochondrial respiratory complexes. Several lines of biochemical evidence suggest that the mitochondrially encoded subunits may form a scaffold during the assembly of respiratory chain complexes that is then joined by nucleus-encoded subunits imported from the cytosol (65, 66).

Unlike the early induction of mitochondrial genome expression factors, the increase in mitochondrial DNA copy number (13) (see Fig. S4 in the supplemental material) and in mitochondrial mass (13) occurs only at late stages of HCMV replication. A similar sequence of events occurs during *Arabidopsis* germination (67). In dry plant seeds, mitochondria appear in typical metabolically inert structures, termed promitochondria, and undergo rapid development and multiplication into mature metabolically active mitochondria following imbibition (the passive uptake of water by the seed) (68, 69). A detailed transcriptional analysis throughout the germination of *Arabidopsis thaliana* seeds revealed that transcripts encoding mtRNA transcription, editing and splicing factors peak early during germination. These are closely followed by transcripts of mitochondrial OXHPOS genes and by mitochondrial translation factors. The expression of nuclear OXHPOS genes lagged behind that of their mitochondrial counterparts (67). It may be that the sequence of events observed during the induction of mitochondrial biogenesis in HCMV-infected cells and in germinating *Arabidopsis* seeds represents an evolutionarily conserved process. Importantly, while mitochondrial translation precedes the increase in mitochondrial mass and mitochondrial DNA copy number during HCMV replication, it appears not to be essential for their induction as neither was blocked by chloramphenicol (13).

What controls mitochondrial biogenesis during HCMV replication? Our data strongly suggest that this is a virally driven process, as it requires active expression of the viral genome and fails to occur using a UV-inactivated virus. We show that mitochondrial expression factors start to accumulate at 24 hpi, suggesting that viral early genes are involved, although the involvement of IE gene expression cannot be excluded. However, neither UL37x1, an IE protein that localizes to mitochondria with early kinetics (21, 22, 70, 71), nor the early β2.7 noncoding RNA (25) (both with documented effects on mitochondrial function) was required for the induction of MRM3 and TFB2M (Fig. 4). The identities of the viral genes that govern this induction remain to be found.

The coordinated induction of mitochondrial transcription and translation factors is likely to be mediated by a cellular regulator, possibly activated by a viral gene product. Seo et al. have previously shown that at 24 h of HCMV infection, viral UL37x1 recruits the cellular protein viperin to mitochondria, where it interferes with fatty acid beta oxidation, causing a 50% reduction in cellular ATP (46). At 72 hpi, however, viperin was relocalized from mitochondria to the cytoplasmic viral assembly compartment (46). This may explain why we have not identified viperin by MS in our mitochondrial preparations at 48 or 60 hpi. We show that the induction of mitochondrial expression factors initiates at 24 hpi. While this induction was independent of UL37x1 (Fig. 4), it remains possible that mitochondrial biogenesis is induced to compensate for an initial perturbation of ATP synthesis which then allows ATP levels to be maintained later during HCMV infection. We have previously reported that following an initial (more subtle) reduction in cellular ATP following HCMV infection of U373 cells, ATP levels persist for at least 5 days postinfection (25). A compensatory increase in mitochondrial biogenesis was reported for multiple respiratory chain dysfunctions (72–74) and was proposed to explain an increase in mitochondrial mass in certain types of tumors (75).

Although several master regulators of mitochondrial biogenesis have been identified (reviewed in reference 76), to our knowledge, there is no transcription factor yet known to specifically regulate the genes encoding mitochondrial transcription and translation proteins. Peroxisome proliferator-activated receptor gamma coactivator 1-alpha (PGC-1α) is a transcriptional coactivator that is induced under physiologic conditions that require mitochondria to produce heat or ATP (76). Although PGC-1α transcription is also induced during HCMV infection of fibroblasts (13), the late kinetics of induction of PGC1α by HCMV suggests that PGC-1α is not the initiator of induction of mitochondrial genome expression we observed during infection. However, PGC-1α may be involved in the late increase in mitochondrial mass. Nuclear respiratory factors 1 and 2 (Nrf1 and 2) are transcription factors that regulate the expression of nucleus-encoded respiratory complex subunits and genes involved in mtDNA transcription and replication, heme biosynthesis, and mitochondrial protein import (76). Nrf2, which also regulates antioxidant genes (77), is induced early during HCMV infection and protects infected cells from oxidative stress (78). Importantly, knockdown of Nrf2 does not cause any inhibition of HCMV growth under nonstressed conditions (78), unlike the reduction observed upon mitochondrial translation block (13) (Fig. 6). Nonetheless, involvement of Nrf2 in HCMV induction of mitochondrial expression factors cannot formally be excluded. Some genes involved in mitochondrial biogenesis are known to be regulated at the level of translation. mTORC inhibitors suppress the translation of mRNAs encoding components of respiratory complex V (ATP synthase), complex I assembly factors, as well as the major mitochondrial transcription factor TFAM and several mitoribosomal proteins (79). Analysis of total and polysome-associated mRNAs from HCMV-infected cells suggested that the translation of several mitoribosomal subunits was induced by the virus (18). Some OXPHOS and mitoribosomal proteins were translationally induced in noninfected cells expressing the HCMV mTORC1 activator UL38, while others, induced by the virus, were not induced by UL38 alone. Our analysis suggests that, at least for MRM3 and TFB2M mitochondrial expression factors (see Fig. S5 in the supplemental material), regulation occurs at the level of transcription.

Our analysis showed that mitochondrial translation is important for HCMV replication, especially under conditions requiring active mitochondrial respiration. Specific inhibition of mitochondrial translation by chloramphenicol or by knockdown of the mitoribosome biogenesis factor MRM3 caused an ~1.5-log reduction in virus titers in galactose-fed cells (Fig. 6A). This inhibition of viral growth by chloramphenicol could not be rescued by the addition of uridine (Fig. 6A), previously shown to recover cell growth arrest by chloramphenicol (61). Taken together, these findings suggest that mitochondrial translation is important to keep mitochondria bioenergetically active during viral replication.

How do other viruses affect mitochondrial biogenesis? Rubella virus infection causes a significant increase in the activity of mitochondrial respiratory complexes II and III, as well as an increase in mitochondrial membrane potential and cellular ATP levels (80, 81). Increases in mitochondrial membrane potential and ATP also occur during persistent infection with measles virus (82). Similar to HCMV, these viruses also have protracted life cycles that may require prolonged maintenance of mitochondrial activity. Of note, among their many functions, interferons have been shown to inhibit mitochondrial gene expression (83–86), supporting the notion that mitochondrial function is supportive of viral replication. In contrast, many viruses with a short replication cycle interfere with mitochondrial activity (87). This is well demonstrated for HSV-1 and HSV-2, which unlike HCMV, have a much shorter replication cycle, that in culture ends with abrupt cytolysis of infected cells. Both HSV-1 infection and HSV-2 infection result in reduced levels of cellular ATP and mitochondrial membrane potential at late stages of HEp-2 infection (88). Similarly, the HSV-1 US3 protein is known to induce inhibition of mitochondrial respiration in HeLa cells (89). Moreover, in contrast to the induction of mtDNA synthesis by HCMV, HSV-1 encodes a mitochondrially targeted nuclease, UL12.5, which degrades mtDNA early during HSV-1 infection (90). Although the importance of mtDNA elimination for HSV-1 replication is not understood (90), it is clear that HSV-1 does not require active and proliferating mitochondria, apparently quite unlike HCMV. The importance of mitochondrial integrity as a cue for an immune response has recently become clear. mtDNA can serve as a stress signal both within and outside the cell. mtDNA released from cells induces inflammation by activation of polymorphonuclear neutrophils (PMNs) through CpG-Toll-like receptor 9 (TLR9) interactions (91). Within cells, mtDNA released from mitochondria during apoptosis is sensed by the cytosolic cGAS DNA sensor and leads to the induction of type I interferons (92–94). It will be interesting to determine whether, in addition to its metabolic and bioenergetic functions, induction of mitochondrial biogenesis is important for keeping HCMV-infected cells immunologically silent.

## MATERIALS AND METHODS

### Cells and viruses.

Primary human fetal foreskin fibroblast cells (HFFF2) and U373 cells were grown in Dulbecco’s modified Eagle’s medium (DMEM) supplemented with 10% (vol/vol) heat-inactivated fetal bovine serum (FBS), 100 µg/ml penicillin-streptomycin, and 2 mM l-glutamine at 37°C in 5% CO_2_. The HCMV strain Merlin (95) was used in all experiments, except if stated otherwise. The Towne-BAC (bacterial artificial chromosome) ΔUL37x1 mutant HCMV and its parental strain (96) were a kind gift from E. Mocarski (Emory University School of Medicine, Atlanta, GA). The Merlin BAC-derived clone was used for the construction of a Δβ2.7 strain by recombineering (see Text S1 in the supplemental material). For the SILAC experiment, media collected from HCMV (Merlin)-infected cells were spun at 13,530 *g* (average) for 2 h at 4°C to pellet viruses. Pellets were then resuspended in SILAC Minimum Essential Medium (lacking lysine and arginine) supplemented with 10% dialyzed FBS.

### Virus infection.

Cells seeded 1 day prior to infection were inoculated with HCMV at a multiplicity of infection (MOI) of 5 for 2 h unless indicated differently and washed once in medium. Where indicated, cells were incubated with PFA at 300 µg/ml, chloramphenicol at 50 or 100 µg/ml, and EtBr at 40 or 80 ng/ml from the time of infection onwards. For infection of the ΔUL37x1 mutant and its parental strain (where viral titers were low), we applied a spin infection protocol, in which plated cells were spun with virus for 30 min at 730 g at room temperature followed by 1 h of incubation in a humidified CO_2_ incubator.

### SILAC.

U373 cells were cultured in SILAC DMEM supplemented with 10% dialyzed FBS (dFBS), 100 µg/ml penicillin-streptomycin, and 2 mM l-glutamine. SILAC medium was supplemented with either “light” (Arg 0, Lys 0 [Sigma]), “medium” (Arg 6, Lys 4, [Cambridge Isotope Laboratories]), or “heavy” (Arg 10, Lys 8 [Cambridge Isotope Laboratories]) amino acids at 50 mg/liter and l-proline at 280 mg/liter. Incorporation of “heavy” label was >98% for both arginine and lysine-containing peptides. Cells were propagated for 7 doublings in SILAC medium to enable incorporation of the labeled amino acids into cellular proteins. Twenty-four hours prior to infection, 75-cm^2^ flasks were seeded with 3.2 × 10^6^ cells. Two flasks were established for each SILAC medium (“light,” “medium,” and “heavy”). Cells were sequentially infected at an MOI of 3 with HCMV strain Merlin (prepared in SILAC DMEM). Mock-infected cells (“heavy”) were inoculated with conditioned medium from HFFF2 cultured cells. Forty-eight-hour (“light”) and 60-h (“medium”) infections were staggered such that all flasks were harvested simultaneously. Infection efficiency was monitored using HCMV IE1 immunostaining.

### Mitochondrial isolation.

Mitochondria were affinity purified using superparamagnetic microbeads conjugated to anti-TOM22 antibodies (Miltenyi Biotec) (see Text S1 in the supplemental material).

### Mass spectrometry and data analysis.

Mass spectrometric analysis, database searching, and data processing were performed as described previously (97). Briefly, for the three-label experiment (Fig. 1; see Table S1A in the supplemental material), mitochondrial protein extracts were separated by gel electrophoresis and divided into 24 gel slices followed by in-gel trypsin digestion. Liquid chromatography-tandem MS (LC-MS/MS) was performed using a NanoAcquity ultraperformance liquid chromatograph (uPLC) (Waters, Milford, MA) coupled to an LTQ-OrbiTrap XL (Thermo Scientific, Tampa, FL). Peptides were eluted using a gradient rising to 25% MeCN by 70 min and 45% MeCN by 80 min. MS data were acquired between 300 and 2,000 *m*/*z* at 60,000 full width at half-maximum (fwhm). Collision-induced dissociation (CID) spectra were acquired in the LTQ device with MS/MS switching operating in a top-6 data-dependent acquisition (DDA) fashion triggered at 500 counts.

For the two-label biological repeat (see Fig. S2B and Table S1B in the supplemental material), mitochondrial protein extracts were dialyzed and trypsinized on a column as described previously (97). Eluted peptides were fractionated off-line by high-pH reverse-phase high-performance liquid chromatography (HpRP-HPLC) using a Dionex UltiMate 3000 powered by an ICS-3000 SP pump with an Agilent Zorbax Extend-C_18_ column (4.6 mm by 250 mm, 5-µm particle size). Mobile phases (H_2_O plus 0.1% NH_4_OH or MeCN plus 0.1% NH_4_OH) were adjusted to pH 10.5 with the addition of formic acid, and peptides were resolved using a linear 40-min 0.1% to 40% MeCN gradient over 40 min at a flow rate of 400 µl/min and a column temperature of 15°C. Eluting peptides were collected in 15-s fractions. LC-MS/MS was performed on 42 combined fractions as described above but with a gradient rising to 45% MeCN by 100 min and MS/MS triggered at 1,000 counts.

Raw data files were processed as previously described (97) using MaxQuant version 1.3.0.5 (98) and a merged *Homo sapiens*/HCMV strain Merlin UniProt database (downloaded 9/5/14). Gene ontology cellular compartment (GOCC) terms were added using the UniProt database (99).

### Pathway analysis.

The Database for Annotation, Visualization and Integrated Discovery (DAVID) was used to determine Gene Ontology biological processes (GOTERM_BP) and KEGG pathway enrichment (28). The groups of virus-induced downregulated and upregulated proteins were searched against a background of all proteins quantified within the relevant experiment.

### Analysis of the mitochondrial ribosome profile on density gradients.

Total cell lysates in (50 mM Tris-HCl, 150 mM NaCl, 1% Triton X-100, supplemented with protease inhibitor cocktail [Roche]) were loaded on a linear sucrose gradient (2 ml 10 to 30% [wt/vol]) in a mixture of 50 mM Tris-HCl (pH 7.2), 10 mM Mg(OAc)_2_, 40 mM NH_4_Cl, and 25 mM KCl and centrifuged for 2 h 15 min at 100,000 × *g*_max_ at 4°C (39,000 rpm [Beckman Coulter TLS-55 rotor]). Twenty fractions (100 µl each) were collected, and 10-µl aliquots of the first 17 fractions containing mitoribosomal subunits and assembled mitoribosomes were analyzed by WB.

### Mitochondrial translation analysis.

Mock-infected and HCMV-infected cells were incubated for 30 min in l-methionine-free DMEM supplemented with 10% dialyzed fetal bovine serum (dFBS) and emetine (100 µg/ml) to block cytosolic translation and 250 µCi/ml [^35^S]methionine (EasyTag l-[^35^S]methionine [PerkinElmer, Waltham, MA]). For total cellular translation, 25 µCi/ml was used without emetine. After labeling, cells were washed twice with excess phosphate-buffered saline (PBS) before lysis with cold radioimmunoprecipitation assay (RIPA) buffer supplemented with complete protease inhibitor cocktail (Sigma-Aldrich). Insoluble material was removed from the lysate by centrifugation for 10 min at 10,000 × *g* at 4^ο^C. Equal amounts of protein were spotted onto Whatman filter papers in 4 replicates. After drying, two replicates were used to measure the total counts per minute ([^35^S]Met incorporated into proteins + [^35^S]Met taken up by the cells yet not incorporated), while two replicates were TCA precipitated to measure the acid-insoluble portion ([^35^S]Met incorporated into proteins only) by incubation of 20 min in cold 10% TCA, boiling for 15 min in 5% TCA, and washing in 5% TCA followed by washing in 95% ethanol and drying. Radioactive counts were measured after addition of Ultima Gold scintillation liquid (PerkinElmer) using a Tri-Carb 1500 liquid scintillation analyzer (PerkinElmer). Translation was calculated as TCA-precipitated counts/total counts. For autoradiography analysis, lysates were separated on 4 to 12% Bis-Tris Plus PAGE, after which the gel was stained with Coomassie brilliant blue G-250 (to confirm equal loading), dried, exposed to a phosphorimager screen, and visualized using a FLA5100 scanner (Fujiimager, Tilburg, The Netherlands).

### Analysis of the stability of mitochondrially encoded proteins.

Mock-infected or HCMV-infected cells at 24 hpi were treated with chloramphenicol (50 µg/ml) to block mitochondrial translation. Cell extracts were made after 0, 6, 12, 24, and 30 h of addition of chloramphenicol and analyzed by WB for mt-CO2, mt-CO1, and glyceraldehyde-3-phosphate dehydrogenase (GAPDH) as a loading control.

### Effect of chloramphenicol treatment on viral titers.

Cells were infected with HCMV (Merlin) at an MOI of 5 for 1 h, washed once, and refreshed with DMEM supplemented with 10% dFBS, penicillin/streptomycin, and 2 mM l-glutamine (DMEM-10dFBS) and 5 mM glucose. At 24 hpi, cells were washed and refreshed with DMEM-10dFBS medium containing either 5 mM glucose or 5 mM galactose (galactose-DMEM-10dFBS) with or without the addition of 0.2 mM uridine. Each medium was either left untreated or treated with chloramphenicol (50 µg/ml) to block mitochondrial translation. At 5 dpi, media were collected from cells and kept at −80°C until assayed for determination of viral titers (see Text S1 in the supplemental material). Cells were tested for propidium iodide exclusion to determine viability.

### siRNA knockdown of MRM3.

RNA interference (RNAi) duplexes (see Text S1 in the supplemental material) were delivered to 10^5^ cells in 12-well culture plates 1 day prior to infection with HCMV using oligofectamine transfection reagent (Life Technologies). Infection was carried out at an MOI of 3 in penicillin-streptomycin-free DMEM-glucose. After 24 h of infection, cells were shifted to galactose-DMEM-10dFBS. After 5 days of infection, media were collected from cells and spun at 800 g for 10 min at 18°C. Supernatants were kept frozen at −80°C until assayed for viral titers. The experiment included three transfection replicates and two biological repeats. Of the three replicate wells, one well was harvested for WB analysis, while the remaining two wells were used to determine cell viability by trypan blue exclusion using a Countess automated cell counter (Life Technologies).

## SUPPLEMENTAL MATERIAL

Text S1  Supplemental experimental procedures. Download Text S1, DOCX file, 0.03 MB

Figure S1  Work flow of SILAC-MS analysis of the mitochondrial proteome following HCMV infection. U373 cells were cultured for 7 passages in “light,” “heavy,” or “medium” SILAC-DMEM. Cells were infected at an MOI of 3 with HCMV strain Merlin. The 48-h and 60-h infections were staggered such that all flasks were harvested simultaneously. Prior to mitochondrial isolation, the 3 cell populations were mixed. Cells were then homogenized, and mitochondria were isolated by affinity purification using superparamagnetic microbeads conjugated to anti-TOM22 antibodies. Purified mitochondria were lysed, and extracted proteins were separated by SDS-PAGE and trypsin digested. Peptides were eluted and analyzed by LC-MS/MS Download Figure S1, PDF file, 0.2 MB

Figure S2  (A) Comparison of the mitochondrial proteome of U373 cells between 48 and 60 h of HCMV infection. Shown is a scatter plot representing the fold change in “heavy” (mock)/“medium” (HCMV 60 hpi) (*y* axis) compared to “heavy” (mock)/“light” (HCMV 48 hpi) (*x* axis), with both axes displayed using a log_2_ scale. The changes in abundance of mitochondrially associated proteins relative to those in mock-infected cells were nearly identical after 48 and 60 h of HCMV infection. (B) An independent biological repeat of the SILAC experiment shows similar changes in the mitochondrial proteome following HCMV infection. Shown is a scatter plot representing the fold change in “heavy” (mock)/“light” (HCMV 48 hpi) in two independent biological repeats, with both axes displayed using a log_2_ scale. Protein ratios show a high degree of correlation between experiments. (C) WB measurements correlate with SILAC data. The fold changes in abundance of mitochondrial proteins at 48 hpi presented in Fig. 2 were assessed by densitometric analysis and compared to the values obtained in the SILAC-MS analysis (average of values from Table S1A and S1B). Download Figure S2, PDF file, 1.3 MB

Figure S3  (A) Proteins with high mTP scores are enriched in infected cells. TargetP scores were plotted against log (H/L ratio) for the whole UniProt database (a) and against the UniProt database of mitochondrial proteins only (annotated by Mitocarta [b]). Proteins with a negative log ratio were relatively enriched in infected cells, which were labeled with light amino acids (red arrow). The significance of difference in the average log (ratio) between populations defined by TargetP 0 to 0.1 and 0.9 to 1 or TargetP 0 to 0.2 and 0.8 to 1 was calculated using a 2-tailed *t* test (2 samples, unequal variance). (B) Fold change measurements were not biased by differences in total amounts of heavy- and light-labeled mitochondrial proteins. Raw MS files were searched using MaxQuant, and fold changes in protein abundance at 48 hpi were calculated, employing 3 databases: a, all human proteins; b, all human proteins with mitochondrial proteins removed; c, all mitochondrial proteins only (annotated by Mitocarta). Pairwise comparison of ratios for data searched against databases b versus a and c versus a are presented. Download Figure S3, PDF file, 1.2 MB

Figure S4  mtDNA is upregulated at late stages of HCMV replication. HFFF2 cells were either mock infected or infected with HCMV at an MOI of 5 for 24, 48, or 72 h. Total DNA was extracted from cells, and the relative expression of mtDNA was determined using quantitative PCR (qPCR). Column charts show the fold change in mtDNA compared to that in mock-infected cells at 24 hpi. Error bars represent standard errors from two biological repeats. Download Figure S4, PDF file, 0.1 MB

Figure S5  TFB2M and MRM3 are induced at the level of mRNA following HCMV infection. HFFF2 cells were either mock infected or infected with HCMV at an MOI of 5 for 48 h. Total RNA was extracted from cells, and the relative expression of TFB2M and MRM3 mRNAs was determined using reverse transcription-quantitative PCR (RT-qPCR) using *GAPDH* and *POLR2L* as the reference genes. Column charts show the fold change in mRNA expression in HCMV-infected over mock-infected cells. Error bars represent standard errors from two biological repeats. Download Figure S5, PDF file, 0.1 MB

Figure S6  (A) Induction of mitochondrially encoded proteins by HCMV was blocked by chloramphenicol. HFFF2 cells were either mock infected or infected with HCMV at an MOI of 5. After 2 h, the inoculum was washed and cells were refreshed with untreated medium (UT) or with medium containing chloramphenicol to block mitochondrial translation. Cells were harvested at 48 hpi, and lysates were processed and analyzed by WB. (B) mt-CO2 was induced in infected U373 cells only at 72 hpi. HFFF2 cells were either mock infected or infected with HCMV for 48 or 72 h. Cells were harvested and lysates were processed and analyzed by WB. The charts in panels A and B show the fold changes in protein abundance after viral infection. (C) Blocking cytosolic translation abrogated mitochondrial translation in mock-infected but not in HCMV-infected cells. Mock-infected and HCMV-infected cells were radiolabeled with [^35^S]methionine either with or without 4 h of pretreatment with emetine (which blocks cytosolic translation). Total cell lysates were separated by 15% SDS-PAGE. Equal loading of the gels was confirmed by staining the gel with Coomassie brilliant blue G-250. Dried gels were exposed to phosphorimager plates and scanned. (D) Mitochondrially encoded proteins are stabilized by HCMV infection. Mock-infected or HCMV-infected HFFF2 cells were treated with chloramphenicol at 24 hpi for the times indicated. Cell lysates were analyzed by WB. Graphs show the fold change in mt-CO1 and mt-CO2 over time relative to *t* = 0 (addition of chloramphenicol) normalized to GAPDH. (E) Pretreatment with chloramphenicol reduces viral growth on glucose-fed cells. Mock-infected or HCMV-infected HFFF2 cells were grown in DMEM supplemented with 5 mM glucose (as described in the legend to Fig. 6A) and were either left untreated or treated with chloramphenicol (50 µg/ml) either 24 h prior to or 24 h after HCMV infection**.** At 3 dpi, media were collected from cells, and released virus titers were quantified using TCID_50_. Error bars represent the standard errors from two experiments with two replications each. *, *P* < 0.05 (unpaired *t* test with Welch’s correction). Download Figure S6, PDF file, 1.7 MB

Table S1  Quantitation of mitochondrially associated proteins upon HCMV infection. (A) Triple-labeling SILAC experiment. (B) Double-labeling SILAC experiment. Table S1, XLSX file, 0.3 MB

Table S2  (A) GO biological processes enriched within virus-downregulated proteins. (B) KEGG pathways enriched within virus-upregulated proteins. Table S2, XLSX file, 0.01 MB

Table S3  Quantitation of OXPHOS subunits and assembly factors upon HCMV infection. Table S3, XLSX file, 0.02 MB

## References

[1] Boppana SB, Britt WJ 2013 Synopsis of clinical aspects of human cytomegalovirus disease, p 1–25. *In* Reddehase MJ (ed), Cytomegaloviruses: from molecular pathogenesis to intervention. Caister Academic Press, Norfolk, United Kingdom.

[2] Munger J, Bajad SU, Coller HA, Shenk T, Rabinowitz JD 2006 Dynamics of the cellular metabolome during human cytomegalovirus infection. PLOS Pathog 2:e132. doi:10.1371/journal.ppat.0020132. 17173481 PMC1698944

[3] Vastag L, Koyuncu E, Grady SL, Shenk TE, Rabinowitz JD 2011 Divergent effects of human cytomegalovirus and herpes simplex virus-1 on cellular metabolism. PLOS Pathog 7:e1002124. doi:10.1371/journal.ppat.1002124. 21779165 PMC3136460

[4] Rabinowitz JD, Shenk T, Reddehase M 2013 Human cytomegalovirus metabolomics, p 59–67. *In* Reddehase MJ (ed), Cytomegaloviruses: from molecular pathogenesis to intervention. Caister Academic Press, Norfolk, United Kingdom.

[5] Yu Y, Clippinger AJ, Alwine JC 2011 Viral effects on metabolism: changes in glucose and glutamine utilization during human cytomegalovirus infection. Trends Microbiol 19:360–367. doi:10.1016/j.tim.2011.04.002. 21570293 PMC3130066

[6] Warburg O 1956 On the origin of cancer cells. Science 123:309–314. doi:10.1126/science.123.3191.309. 13298683

[7] Landini MP 1984 Early enhanced glucose uptake in human cytomegalovirus-infected cells. J Gen Virol 65:1229–1232. doi:10.1099/0022-1317-65-7-1229. 6086816

[8] Yu Y, Maguire TG, Alwine JC 2011 Human cytomegalovirus activates glucose transporter 4 expression to increase glucose uptake during infection. J Virol 85:1573–1580. doi:10.1128/JVI.01967-10. 21147915 PMC3028904

[9] Munger J, Bennett BD, Parikh A, Feng XJ, McArdle J, Rabitz HA, Shenk T, Rabinowitz JD 2008 Systems-level metabolic flux profiling identifies fatty acid synthesis as a target for antiviral therapy. Nat Biotechnol 26:1179–1186. doi:10.1038/nbt.1500. 18820684 PMC2825756

[10] Chambers JW, Maguire TG, Alwine JC 2010 Glutamine metabolism is essential for human cytomegalovirus infection. J Virol 84:1867–1873. doi:10.1128/JVI.02123-09. 19939921 PMC2812398

[11] Crabtree HG 1929 Observations on the carbohydrate metabolism of tumours. Biochem J 23:536–545. doi:10.1042/bj0230536. 16744238 PMC1254097

[12] De Deken RH 1966 The Crabtree effect: a regulatory system in yeast. J Gen Microbiol 44:149–156. doi:10.1099/00221287-44-2-149. 5969497

[13] Kaarbø M, Ager-Wick E, Osenbroch PØ, Kilander A, Skinnes R, Müller F, Eide L 2011 Human cytomegalovirus infection increases mitochondrial biogenesis. Mitochondrion 11:935–945. doi:10.1016/j.mito.2011.08.008. 21907833

[14] West AP, Shadel GS, Ghosh S 2011 Mitochondria in innate immune responses. Nat Rev Immunol 11:389–402. doi:10.1038/nri2975. 21597473 PMC4281487

[15] Calvo SE, Mootha VK 2010 The mitochondrial proteome and human disease. Annu Rev Genomics Hum Genet 11:25–44. doi:10.1146/annurev-genom-082509-141720. 20690818 PMC4397899

[16] Furukawa T, Sakuma S, Plotkin SA 1976 Human cytomegalovirus infection of WI-38 cells stimulates mitochondrial DNA synthesis. Nature 262:414–416. doi:10.1038/262414a0. 183130

[17] Hertel L, Mocarski ES 2004 Global analysis of host cell gene expression late during cytomegalovirus infection reveals extensive dysregulation of cell cycle gene expression and induction of pseudomitosis independent of US28 function. J Virol 78:11988–12011. doi:10.1128/JVI.78.21.11988-12011.2004. 15479839 PMC523267

[18] McKinney C, Zavadil J, Bianco C, Shiflett L, Brown S, Mohr I 2014 Global reprogramming of the cellular translational landscape facilitates cytomegalovirus replication. Cell Rep 6:9–17. doi:10.1016/j.celrep.2013.11.045. 24373965 PMC3975909

[19] Weekes MP, Tomasec P, Huttlin EL, Fielding CA, Nusinow D, Stanton RJ, Wang EC, Aicheler R, Murrell I, Wilkinson GW, Lehner PJ, Gygi SP 2014 Quantitative temporal viromics: an approach to investigate host-pathogen interaction. Cell 157:1460–1472. doi:10.1016/j.cell.2014.04.028. 24906157 PMC4048463

[20] Zhang A, Williamson CD, Wong DS, Bullough MD, Brown KJ, Hathout Y, Colberg-Poley AM 2011 Quantitative proteomic analyses of human cytomegalovirus-induced restructuring of endoplasmic reticulum-mitochondrial contacts at late times of infection. Mol Cell Proteomics 10:M111.009936. doi:10.1074/mcp.M111.009936. PMC3205871 21742798

[21] Goldmacher VS, Bartle LM, Skaletskaya A, Dionne CA, Kedersha NL, Vater CA, Han JW, Lutz RJ, Watanabe S, Cahir McFarland ED, Kieff ED, Mocarski ES, Chittenden T 1999 A cytomegalovirus-encoded mitochondria-localized inhibitor of apoptosis structurally unrelated to Bcl-2. Proc Natl Acad Sci U S A 96:12536–12541. doi:10.1073/pnas.96.22.12536. 10535957 PMC22976

[22] McCormick AL, Smith VL, Chow D, Mocarski ES 2003 Disruption of mitochondrial networks by the human cytomegalovirus UL37 gene product viral mitochondrion-localized inhibitor of apoptosis. J Virol 77:631–641. doi:10.1128/JVI.77.1.631-641.2003. 12477866 PMC140587

[23] Palmer CS, Osellame LD, Stojanovski D, Ryan MT 2011 The regulation of mitochondrial morphology: intricate mechanisms and dynamic machinery. Cell Signal 23:1534–1545. doi:10.1016/j.cellsig.2011.05.021. 21683788

[24] Landini MP, Rugolo M 1984 Increased accumulation of a lipophilic cation (tetraphenylphosphonium) in human embryo fibroblasts after infection with cytomegalovirus. J Gen Virol 65:2269–2272. doi:10.1099/0022-1317-65-12-2269. 6096498

[25] Reeves MB, Davies AA, McSharry BP, Wilkinson GW, Sinclair JH 2007 Complex I binding by a virally encoded RNA regulates mitochondria-induced cell death. Science 316:1345–1348. doi:10.1126/science.1142984. 17540903

[26] Lee K-W, Okot-Kotber C, LaComb JF, Bogenhagen DF 2013 Mitochondrial ribosomal RNA (rRNA) methyltransferase family members are positioned to modify nascent rRNA in foci near the mitochondrial DNA nucleoid. J Biol Chem 288:31386–31399. doi:10.1074/jbc.M113.515692. 24036117 PMC3829452

[27] Rorbach J, Boesch P, Gammage PA, Nicholls TJ, Pearce SF, Patel D, Hauser A, Perocchi F, Minczuk M 2014 MRM2 and MRM3 are involved in biogenesis of the large subunit of the mitochondrial ribosome. Mol Biol Cell 25:2542–2555. doi:10.1091/mbc.E14-01-0014. 25009282 PMC4148245

[28] Huang DW, Sherman BT, Lempicki RA 2009 Systematic and integrative analysis of large gene lists using DAVID bioinformatics resources. Nat Protoc 4:44–57. doi:10.1038/nprot.2008.211. 19131956

[29] Poncet D, Pauleau AL, Szabadkai G, Vozza A, Scholz SR, Le Bras M, Brière JJ, Jalil A, Le Moigne R, Brenner C, Hahn G, Wittig I, Schägger H, Lemaire C, Bianchi K, Souquère S, Pierron G, Rustin P, Goldmacher VS, Rizzuto R, Palmieri F, Kroemer G 2006 Cytopathic effects of the cytomegalovirus-encoded apoptosis inhibitory protein vMIA. J Cell Biol 174:985–996. doi:10.1083/jcb.200604069. 16982800 PMC2064390

[30] Sharon-Friling R, Goodhouse J, Colberg-Poley AM, Shenk T 2006 Human cytomegalovirus pUL37x1 induces the release of endoplasmic reticulum calcium stores. Proc Natl Acad Sci U S A 103:19117–19122. doi:10.1073/pnas.0609353103. 17135350 PMC1748185

[31] Stanton RJ, Prod’homme V, Purbhoo MA, Moore M, Aicheler RJ, Heinzmann M, Bailer SM, Haas J, Antrobus R, Weekes MP, Lehner PJ, Vojtesek B, Miners KL, Man S, Wilkie GS, Davison AJ, Wang EC, Tomasec P, Wilkinson GW 2014 HCMV pUL135 remodels the actin cytoskeleton to impair immune recognition of infected cells. Cell Host Microbe 16:201–214. doi:10.1016/j.chom.2014.07.005. 25121749 PMC4150922

[32] Qattan AT, Radulovic M, Crawford M, Godovac-Zimmermann J 2012 Spatial distribution of cellular function: the partitioning of proteins between mitochondria and the nucleus in MCF7 breast cancer cells. J Proteome Res 11:6080–6101. doi:10.1021/pr300736v. 23051583 PMC4261608

[33] Giegé P, Heazlewood JL, Roessner-Tunali U, Millar AH, Fernie AR, Leaver CJ, Sweetlove LJ 2003 Enzymes of glycolysis are functionally associated with the mitochondrion in Arabidopsis cells. Plant Cell 15:2140–2151. doi:10.1105/tpc.012500. 12953116 PMC181336

[34] Wojtera-Kwiczor J, Groß F, Leffers HM, Kang M, Schneider M, Scheibe R 2012 Transfer of a redox-signal through the cytosol by redox-dependent microcompartmentation of glycolytic enzymes at mitochondria and actin cytoskeleton. Front Plant Sci 3:284. doi:10.3389/fpls.2012.00284. 23316205 PMC3540817

[35] Graham JW, Williams TC, Morgan M, Fernie AR, Ratcliffe RG, Sweetlove LJ 2007 Glycolytic enzymes associate dynamically with mitochondria in response to respiratory demand and support substrate channeling. Plant Cell 19:3723–3738. doi:10.1105/tpc.107.053371. 17981998 PMC2174870

[36] Cao W, Liu N, Tang S, Bao L, Shen L, Yuan H, Zhao X, Lu H 2008 Acetyl-coenzyme A acyltransferase 2 attenuates the apoptotic effects of BNIP3 in two human cell lines. Biochim Biophys Acta 1780:873–880. doi:10.1016/j.bbagen.2008.02.007. 18371312

[37] Trilling M, Hengel H 2013 Cytomegaloviruses and interferons, p 278 *In* Reddehase MJ (ed), Cytomegaloviruses: from molecular pathogenesis to intervention. Caister Academic Press, Norfolk, United Kingdom.

[38] Scott I 2009 Degradation of RIG-I following cytomegalovirus infection is independent of apoptosis. Microbes Infect 11:973–979. doi:10.1016/j.micinf.2009.07.001. 19591957 PMC2741008

[39] Unterholzner L, Keating SE, Baran M, Horan KA, Jensen SB, Sharma S, Sirois CM, Jin T, Latz E, Xiao TS, Fitzgerald KA, Paludan SR, Bowie AG 2010 IFI16 is an innate immune sensor for intracellular DNA. Nat Immunol 11:997–1004. doi:10.1038/ni.1932. 20890285 PMC3142795

[40] Gariano GR, Dell’Oste V, Bronzini M, Gatti D, Luganini A, De Andrea M, Gribaudo G, Gariglio M, Landolfo S 2012 The intracellular DNA sensor IFI16 gene acts as restriction factor for human cytomegalovirus replication. PLoS Pathog 8:e1002498. doi:10.1371/journal.ppat.1002498. 22291595 PMC3266931

[41] Yang J, Zhu X, Liu J, Ding X, Han M, Hu W, Wang X, Zhou Z, Wang S 2012 Inhibition of hepatitis B virus replication by phospholipid scramblase 1 in vitro and in vivo. Antiviral Res 94:9–17. doi:10.1016/j.antiviral.2012.01.010. 22342889

[42] Dong B, Zhou Q, Zhao J, Zhou A, Harty RN, Bose S, Banerjee A, Slee R, Guenther J, Williams BR, Wiedmer T, Sims PJ, Silverman RH 2004 Phospholipid scramblase 1 potentiates the antiviral activity of interferon. J Virol 78:8983–8993. doi:10.1128/JVI.78.17.8983-8993.2004. 15308695 PMC506946

[43] Gotoh T, Mori M 1999 Arginase II downregulates nitric oxide (NO) production and prevents NO-mediated apoptosis in murine macrophage-derived RAW 264.7 cells. J Cell Biol 144:427–434. doi:10.1083/jcb.144.3.427. 9971738 PMC2132906

[44] Mistry SK, Zheng M, Rouse BT, Morris SM Jr. 2001 Induction of arginases I and II in cornea during herpes simplex virus infection. Virus Res 73:177–182. doi:10.1016/S0168-1702(00)00243-4. 11172921

[45] Koyuncu E, Purdy JG, Rabinowitz JD, Shenk T 2013 Saturated very long chain fatty acids are required for the production of infectious human cytomegalovirus progeny. PLoS Pathog 9:e1003333. doi:10.1371/journal.ppat.1003333. 23696731 PMC3656100

[46] Seo JY, Yaneva R, Hinson ER, Cresswell P 2011 Human cytomegalovirus directly induces the antiviral protein viperin to enhance infectivity. Science 332:1093–1097. doi:10.1126/science.1202007. 21527675

[47] Seo J-Y, Cresswell P 2013 Viperin regulates cellular lipid metabolism during human cytomegalovirus infection. PLoS Pathog 9:e1003497. doi:10.1371/journal.ppat.1003497. 23935494 PMC3731232

[48] Emanuelsson O, Nielsen H, Brunak S, von Heijne G 2000 Predicting subcellular localization of proteins based on their N-terminal amino acid sequence. J Mol Biol 300:1005–1016. doi:10.1006/jmbi.2000.3903. 10891285

[49] Calvo SE, Clauser KR, Mootha VK 2016 MitoCarta2.0: an updated inventory of mammalian mitochondrial proteins. Nucleic Acids Res 44:D1251–D1257. doi:10.1093/nar/gkv1003. 26450961 PMC4702768

[50] Wredenberg A, Lagouge M, Bratic A, Metodiev MD, Spåhr H, Mourier A, Freyer C, Ruzzenente B, Tain L, Grönke S, Baggio F, Kukat C, Kremmer E, Wibom R, Polosa PL, Habermann B, Partridge L, Park CB, Larsson N-G 2013 MTERF3 regulates mitochondrial ribosome biogenesis in invertebrates and mammals. PLOS Genet 9:e1003178. doi:10.1371/journal.pgen.1003178. 23300484 PMC3536695

[51] Dalla Rosa I, Durigon R, Pearce SF, Rorbach J, Hirst EM, Vidoni S, Reyes A, Brea-Calvo G, Minczuk M, Woellhaf MW, Herrmann JM, Huynen MA, Holt IJ, Spinazzola A 2014 MPV17L2 is required for ribosome assembly in mitochondria. Nucleic Acids Res 42:8500–8515. doi:10.1093/nar/gku513. 24948607 PMC4117752

[52] Metodiev MD, Spåhr H, Loguercio Polosa P, Meharg C, Becker C, Altmueller J, Habermann B, Larsson NG, Ruzzenente B 2014 NSUN4 is a dual function mitochondrial protein required for both methylation of 12S rRNA and coordination of mitoribosomal assembly. PLoS Genet 10:e1004110. doi:10.1371/journal.pgen.1004110. 24516400 PMC3916286

[53] Uchiumi T, Ohgaki K, Yagi M, Aoki Y, Sakai A, Matsumoto S, Kang D 2010 ERAL1 is associated with mitochondrial ribosome and elimination of ERAL1 leads to mitochondrial dysfunction and growth retardation. Nucleic Acids Res 38:5554–5568. doi:10.1093/nar/gkq305. 20430825 PMC2938226

[54] Dennerlein S, Rozanska A, Wydro M, Chrzanowska-Lightowlers ZM, Lightowlers RN 2010 Human ERAL1 is a mitochondrial RNA chaperone involved in the assembly of the 28S small mitochondrial ribosomal subunit. Biochem J 430:551–558. doi:10.1042/BJ20100757. 20604745 PMC2995420

[55] Tibbetts AS, Appling DR 2010 Compartmentalization of mammalian folate-mediated one-carbon metabolism. Annu Rev Nutr 30:57–81. doi:10.1146/annurev.nutr.012809.104810. 20645850

[56] Lagouge M, Mourier A, Lee HJ, Spåhr H, Wai T, Kukat C, Silva Ramos E, Motori E, Busch JD, Siira S, Kremmer E, Filipovska A, Larsson NG 2015 SLIRP regulates the rate of mitochondrial protein synthesis and protects LRPPRC from degradation. PLoS Genet 11:e1005423. doi:10.1371/journal.pgen.1005423. 26247782 PMC4527767

[57] Walsh D, Mohr I 2011 Viral subversion of the host protein synthesis machinery. Nat Rev Microbiol 9:860–875. doi:10.1038/nrmicro2655. 22002165 PMC7097311

[58] Adamson LF, Langeluttig SG, Anast CS 1966 Inhibition by puromycin of amino acid transport by embryonic chick bone. Biochim Biophys Acta 115:355–360. doi:10.1016/0304-4165(66)90435-1. 5943439

[59] Costantino P, Attardi G 1977 Metabolic properties of the products of mitochondrial protein synthesis in HeLa cells. J Biol Chem 252:1702–1711. 838736

[60] Robinson BH, Petrova-Benedict R, Buncic JR, Wallace DC 1992 Nonviability of cells with oxidative defects in galactose medium: a screening test for affected patient fibroblasts. Biochem Med Metab Biol 48:122–126. doi:10.1016/0885-4505(92)90056-5. 1329873

[61] Grégoire M, Morais R, Quilliam MA, Gravel D 1984 On auxotrophy for pyrimidines of respiration-deficient chick embryo cells. Eur J Biochem 142:49–55. doi:10.1111/j.1432-1033.1984.tb08249.x. 6086342

[62] Evers DL, Wang X, Huong SM, Andreoni KA, Huang ES 2005 Inhibition of human cytomegalovirus signaling and replication by the immunosuppressant FK778. Antiviral Res 65:1–12. doi:10.1016/j.antiviral.2004.03.007. 15652966

[63] Towler JC, Ebrahimi B, Lane B, Davison AJ, Dargan DJ 2012 Human cytomegalovirus transcriptome activity differs during replication in human fibroblast, epithelial and astrocyte cell lines. J Gen Virol 93:1046–1058. doi:10.1099/vir.0.038083-0. 22258857 PMC3541802

[64] Amunts A, Brown A, Toots J, Scheres SH, Ramakrishnan V 2015 Ribosome. The structure of the human mitochondrial ribosome. Science 348:95–98. doi:10.1126/science.aaa1193. 25838379 PMC4501431

[65] Lazarou M, McKenzie M, Ohtake A, Thorburn DR, Ryan MT 2007 Analysis of the assembly profiles for mitochondrial- and nuclear-DNA-encoded subunits into complex I. Mol Cell Biol 27:4228–4237. doi:10.1128/MCB.00074-07. 17438127 PMC1900046

[66] Acín-Pérez R, Fernández-Silva P, Peleato ML, Pérez-Martos A, Enriquez JA 2008 Respiratory active mitochondrial supercomplexes. Mol Cell 32:529–539. doi:10.1016/j.molcel.2008.10.021. 19026783

[67] Law SR, Narsai R, Taylor NL, Delannoy E, Carrie C, Giraud E, Millar AH, Small I, Whelan J 2012 Nucleotide and RNA metabolism prime translational initiation in the earliest events of mitochondrial biogenesis during Arabidopsis germination. Plant Physiol 158:1610–1627. doi:10.1104/pp.111.192351. 22345507 PMC3320173

[68] Howell KA, Millar AH, Whelan J 2006 Ordered assembly of mitochondria during rice germination begins with pro-mitochondrial structures rich in components of the protein import apparatus. Plant Mol Biol 60:201–223. doi:10.1007/s11103-005-3688-7. 16429260

[69] Logan DC, Millar AH, Sweetlove LJ, Hill SA, Leaver CJ 2001 Mitochondrial biogenesis during germination in maize embryos. Plant Physiol 125:662–672. doi:10.1104/pp.125.2.662. 11161024 PMC64868

[70] Mavinakere MS, Colberg-Poley AM 2004 Dual targeting of the human cytomegalovirus UL37 exon 1 protein during permissive infection. J Gen Virol 85:323–329. doi:10.1099/vir.0.19589-0. 14769889

[71] Bozidis P, Williamson CD, Wong DS, Colberg-Poley AM 2010 Trafficking of UL37 proteins into mitochondrion-associated membranes during permissive human cytomegalovirus infection. J Virol 84:7898–7903. doi:10.1128/JVI.00885-10. 20504938 PMC2897597

[72] Cámara Y, Asin-Cayuela J, Park CB, Metodiev MD, Shi Y, Ruzzenente B, Kukat C, Habermann B, Wibom R, Hultenby K, Franz T, Erdjument-Bromage H, Tempst P, Hallberg BM, Gustafsson CM, Larsson NG 2011 MTERF4 regulates translation by targeting the methyltransferase NSUN4 to the mammalian mitochondrial ribosome. Cell Metab 13:527–539. doi:10.1016/j.cmet.2011.04.002. 21531335

[73] Metodiev MD, Lesko N, Park CB, Cámara Y, Shi Y, Wibom R, Hultenby K, Gustafsson CM, Larsson NG 2009 Methylation of 12S rRNA is necessary for in vivo stability of the small subunit of the mammalian mitochondrial ribosome. Cell Metab 9:386–397. doi:10.1016/j.cmet.2009.03.001. 19356719

[74] Wredenberg A, Wibom R, Wilhelmsson H, Graff C, Wiener HH, Burden SJ, Oldfors A, Westerblad H, Larsson NG 2002 Increased mitochondrial mass in mitochondrial myopathy mice. Proc Natl Acad Sci U S A 99:15066–15071. doi:10.1073/pnas.232591499. 12417746 PMC137544

[75] Nisoli E, Carruba MO 2006 Nitric oxide and mitochondrial biogenesis. J Cell Sci 119:2855–2862. doi:10.1242/jcs.03062. 16825426

[76] Kelly DP, Scarpulla RC 2004 Transcriptional regulatory circuits controlling mitochondrial biogenesis and function. Genes Dev 18:357–368. doi:10.1101/gad.1177604. 15004004

[77] Piantadosi CA, Suliman HB 2012 Redox regulation of mitochondrial biogenesis. Free Radic Biol Med 53:2043–2053. doi:10.1016/j.freeradbiomed.2012.09.014. 23000245 PMC3604744

[78] Lee J, Koh K, Kim YE, Ahn JH, Kim S 2013 Upregulation of Nrf2 expression by human cytomegalovirus infection protects host cells from oxidative stress. J Gen Virol 94:1658–1668. doi:10.1099/vir.0.052142-0. 23580430

[79] Morita M, Gravel SP, Chénard V, Sikström K, Zheng L, Alain T, Gandin V, Avizonis D, Arguello M, Zakaria C, McLaughlan S, Nouet Y, Pause A, Pollak M, Gottlieb E, Larsson O, St-Pierre J, Topisirovic I, Sonenberg N 2013 mTORC1 controls mitochondrial activity and biogenesis through 4E-BP-dependent translational regulation. Cell Metab 18:698–711. doi:10.1016/j.cmet.2013.10.001. 24206664

[80] Claus C, Schönefeld K, Hübner D, Chey S, Reibetanz U, Liebert UG 2013 Activity increase in respiratory chain complexes by rubella virus with marginal induction of oxidative stress. J Virol 87:8481–8492. doi:10.1128/JVI.00533-13. 23720730 PMC3719815

[81] Claus C, Chey S, Heinrich S, Reins M, Richardt B, Pinkert S, Fechner H, Gaunitz F, Schäfer I, Seibel P, Liebert UG 2011 Involvement of p32 and microtubules in alteration of mitochondrial functions by rubella virus. J Virol 85:3881–3892. doi:10.1128/JVI.02492-10. 21248045 PMC3126120

[82] Takahashi M, Wolf AM, Watari E, Norose Y, Ohta S, Takahashi H 2013 Increased mitochondrial functions in human glioblastoma cells persistently infected with measles virus. Antiviral Res 99:238–244. doi:10.1016/j.antiviral.2013.06.016. 23830853

[83] Kortsaris A, Taylor-Papadimitriou J, Georgatsos JG 1976 Interferon inhibition of protein synthesis by isolated mitochondria. Biochem Biophys Res Commun 68:1317–1322. doi:10.1016/0006-291X(76)90340-5. 1267779

[84] Lewis JA, Huq A, Najarro P 1996 Inhibition of mitochondrial function by interferon. J Biol Chem 271:13184–13190. doi:10.1074/jbc.271.22.13184. 8662694

[85] Shan B, Vazquez E, Lewis JA 1990 Interferon selectively inhibits the expression of mitochondrial genes: a novel pathway for interferon-mediated responses. EMBO J 9:4307–4314. 2176148 10.1002/j.1460-2075.1990.tb07879.x PMC552214

[86] Lou J, Anderson SL, Xing L, Rubin BY 1994 Suppression of mitochondrial mRNA levels and mitochondrial function in cells responding to the anticellular action of interferon. J Interferon Res 14:33–40. doi:10.1089/jir.1994.14.33. 7517985

[87] Claus C, Liebert UG 2014 A renewed focus on the interplay between viruses and mitochondrial metabolism. Arch Virol 159:1267–1277. doi:10.1007/s00705-013-1841-1. 24343264

[88] Murata T, Goshima F, Daikoku T, Inagaki-Ohara K, Takakuwa H, Kato K, Nishiyama Y 2000 Mitochondrial distribution and function in herpes simplex virus-infected cells. J Gen Virol 81:401–406. doi:10.1099/0022-1317-81-2-401. 10644838

[89] Derakhshan M, Willcocks MM, Salako MA, Kass GE, Carter MJ 2006 Human herpesvirus 1 protein US3 induces an inhibition of mitochondrial electron transport. J Gen Virol 87:2155–2159. doi:10.1099/vir.0.81949-0. 16847111

[90] Duguay BA, Saffran HA, Ponomarev A, Duley SA, Eaton HE, Smiley JR 2014 Elimination of mitochondrial DNA is not required for herpes simplex virus 1 replication. J Virol 88:2967–2976. doi:10.1128/JVI.03129-13. 24371054 PMC3958086

[91] Zhang Q, Raoof M, Chen Y, Sumi Y, Sursal T, Junger W, Brohi K, Itagaki K, Hauser CJ 2010 Circulating mitochondrial DAMPs cause inflammatory responses to injury. Nature 464:104–107. doi:10.1038/nature08780. 20203610 PMC2843437

[92] West AP, Khoury-Hanold W, Staron M, Tal MC, Pineda CM, Lang SM, Bestwick M, Duguay BA, Raimundo N, MacDuff DA, Kaech SM, Smiley JR, Means RE, Iwasaki A, Shadel GS 2015 Mitochondrial DNA stress primes the antiviral innate immune response. Nature 520:553–557. doi:10.1038/nature14156. 25642965 PMC4409480

[93] Rongvaux A, Jackson R, Harman CC, Li T, West AP, de Zoete MR, Wu Y, Yordy B, Lakhani SA, Kuan CY, Taniguchi T, Shadel GS, Chen ZJ, Iwasaki A, Flavell RA 2014 Apoptotic caspases prevent the induction of type I interferons by mitochondrial DNA. Cell 159:1563–1577. doi:10.1016/j.cell.2014.11.037. 25525875 PMC4272443

[94] White MJ, McArthur K, Metcalf D, Lane RM, Cambier JC, Herold MJ, van Delft MF, Bedoui S, Lessene G, Ritchie ME, Huang DC, Kile BT 2014 Apoptotic caspases suppress mtDNA-induced STING-mediated type I IFN production. Cell 159:1549–1562. doi:10.1016/j.cell.2014.11.036. 25525874 PMC4520319

[95] Dolan A, Cunningham C, Hector RD, Hassan-Walker AF, Lee L, Addison C, Dargan DJ, McGeoch DJ, Gatherer D, Emery VC, Griffiths PD, Sinzger C, McSharry BP, Wilkinson GW, Davison AJ 2004 Genetic content of wild-type human cytomegalovirus. J Gen Virol 85:1301–1312. doi:10.1099/vir.0.79888-0. 15105547

[96] McCormick AL, Meiering CD, Smith GB, Mocarski ES 2005 Mitochondrial cell death suppressors carried by human and murine cytomegalovirus confer resistance to proteasome inhibitor-induced apoptosis. J Virol 79:12205–12217. doi:10.1128/JVI.79.19.12205-12217.2005. 16160147 PMC1211555

[97] Weekes MP, Antrobus R, Talbot S, Hör S, Simecek N, Smith DL, Bloor S, Randow F, Lehner PJ 2012 Proteomic plasma membrane profiling reveals an essential role for gp96 in the cell surface expression of LDLR family members, including the LDL receptor and LRP6. J Proteome Res 11:1475–1484. doi:10.1021/pr201135e. 22292497 PMC3292266

[98] Cox J, Neuhauser N, Michalski A, Scheltema RA, Olsen JV, Mann M 2011 Andromeda: a peptide search engine integrated into the MaxQuant environment. J Proteome Res 10:1794–1805. doi:10.1021/pr101065j. 21254760

[99] UniProt Consortium 2015 UniProt: a hub for protein information. Nucleic Acids Res 43:D204–D212. doi:10.1093/nar/gku989. 25348405 PMC4384041

[100] Cheng X, Kanki T, Fukuoh A, Ohgaki K, Takeya R, Aoki Y, Hamasaki N, Kang D 2005 PDIP38 associates with proteins constituting the mitochondrial DNA nucleoid. J Biochem 138:673–678. doi:10.1093/jb/mvi169. 16428295

[101] Cavdar Koc E, Burkhart W, Blackburn K, Moseley A, Spremulli LL 2001 The small subunit of the mammalian mitochondrial ribosome. Identification of the full complement of ribosomal proteins present. J Biol Chem 276:19363–19374. doi:10.1074/jbc.M100727200. 11279123

[102] Koc EC, Burkhart W, Blackburn K, Moyer MB, Schlatzer DM, Moseley A, Spremulli LL 2001 The large subunit of the mammalian mitochondrial ribosome. Analysis of the complement of ribosomal proteins present. J Biol Chem 276:43958–43969. doi:10.1074/jbc.M106510200. 11551941

[103] Falkenberg M, Gaspari M, Rantanen A, Trifunovic A, Larsson N-G, Gustafsson CM 2002 Mitochondrial transcription factors B1 and B2 activate transcription of human mtDNA. Nat Genet 31:289–294. doi:10.1038/ng909. 12068295

[104] Kotani T, Akabane S, Takeyasu K, Ueda T, Takeuchi N 2013 Human G-proteins, ObgH1 and Mtg1, associate with the large mitochondrial ribosome subunit and are involved in translation and assembly of respiratory complexes. Nucleic Acids Res 41:3713–3722. doi:10.1093/nar/gkt079. 23396448 PMC3616715

[105] Minczuk M, Piwowarski J, Papworth MA, Awiszus K, Schalinski S, Dziembowski A, Dmochowska A, Bartnik E, Tokatlidis K, Stepien PP, Borowski P 2002 Localisation of the human hSuv3p helicase in the mitochondrial matrix and its preferential unwinding of dsDNA. Nucleic Acids Res 30:5074–5086. doi:10.1093/nar/gkf647. 12466530 PMC137961

[106] Boczonadi V, Smith PM, Pyle A, Gomez-Duran A, Schara U, Tulinius M, Chinnery PF, Horvath R 2013 Altered 2-thiouridylation impairs mitochondrial translation in reversible infantile respiratory chain deficiency. Hum Mol Genet 22:4602–4615. doi:10.1093/hmg/ddt309. 23814040 PMC3889809

[107] Simon M, Richard EM, Wang X, Shahzad M, Huang VH, Qaiser TA, Potluri P, Mahl SE, Davila A, Nazli S, Hancock S, Yu M, Gargus J, Chang R, Al-Sheqaih N, Newman WG, Abdenur J, Starr A, Hegde R, Dorn T, Busch A, Park E, Wu J, Schwenzer H, Flierl A, Florentz C, Sissler M, Khan SN, Li R, Guan MX, Friedman TB, Wu DK, Procaccio V, Riazuddin S, Wallace DC, Ahmed ZM, Huang T 2015 Mutations of human NARS2, encoding the mitochondrial asparaginyl-tRNA synthetase, cause nonsyndromic deafness and Leigh syndrome. PLoS Genet 11:e1005097. doi:10.1371/journal.pgen.1005097. 25807530 PMC4373692

[108] Holzmann J, Frank P, Löffler E, Bennett KL, Gerner C, Rossmanith W 2008 RNase P without RNA: identification and functional reconstitution of the human mitochondrial tRNA processing enzyme. Cell 135:462–474. doi:10.1016/j.cell.2008.09.013. 18984158

[109] Antonicka H, Sasarman F, Nishimura T, Paupe V, Shoubridge EA 2013 The mitochondrial RNA-binding protein GRSF1 localizes to RNA granules and is required for posttranscriptional mitochondrial gene expression. Cell Metab 17:386–398. doi:10.1016/j.cmet.2013.02.006. 23473033

[110] Antonicka H, Shoubridge E 12 2 2015 Mitochondrial RNA granules are centers for posttranscriptional RNA processing and ribosome biogenesis. Cell Rep doi:10.1016/j.celrep.2015.01.030. 25683715

[111] Bonnefond L, Fender A, Rudinger-Thirion J, Giegé R, Florentz C, Sissler M 2005 Toward the full set of human mitochondrial aminoacyl-tRNA synthetases: characterization of AspRS and TyrRS. Biochemistry 44:4805–4816. doi:10.1021/bi047527z. 15779907

[112] Tucker EJ, Hershman SG, Köhrer C, Belcher-Timme CA, Patel J, Goldberger OA, Christodoulou J, Silberstein JM, McKenzie M, Ryan MT, Compton AG, Jaffe JD, Carr SA, Calvo SE, RajBhandary UL, Thorburn DR, Mootha VK 2011 Mutations in MTFMT underlie a human disorder of formylation causing impaired mitochondrial translation. Cell Metab 14:428–434. doi:10.1016/j.cmet.2011.07.010. 21907147 PMC3486727

[113] Villarroya M, Prado S, Esteve JM, Soriano MA, Aguado C, Pérez-Martínez D, Martínez-Ferrandis JI, Yim L, Victor VM, Cebolla E, Montaner A, Knecht E, Armengod ME 2008 Characterization of human GTPBP3, a GTP-binding protein involved in mitochondrial tRNA modification. Mol Cell Biol 28:7514–7531. doi:10.1128/MCB.00946-08. 18852288 PMC2593442

[114] Chujo T, Ohira T, Sakaguchi Y, Goshima N, Nomura N, Nagao A, Suzuki T 2012 LRPPRC/SLIRP suppresses PNPase-mediated mRNA decay and promotes polyadenylation in human mitochondria. Nucleic Acids Res 40:8033–8047. doi:10.1093/nar/gks506. 22661577 PMC3439899

[115] Hipps D, Shiba K, Henderson B, Schimmel P 1995 Operational RNA code for amino acids: species-specific aminoacylation of minihelices switched by a single nucleotide. Proc Natl Acad Sci U S A 92:5550–5552. doi:10.1073/pnas.92.12.5550. 7539919 PMC41733

[116] Korhonen JA, Gaspari M, Falkenberg M 2003 TWINKLE has 5′→3’ DNA helicase activity and is specifically stimulated by mitochondrial single-stranded DNA-binding protein. J Biol Chem 278:48627–48632. doi:10.1074/jbc.M306981200. 12975372

[117] Coenen MJ, Antonicka H, Ugalde C, Sasarman F, Rossi R, Heister JG, Newbold RF, Trijbels FJ, van den Heuvel LP, Shoubridge EA, Smeitink JA 2004 Mutant mitochondrial elongation factor G1 and combined oxidative phosphorylation deficiency. N Engl J Med 351:2080–2086. doi:10.1056/NEJMoa041878. 15537906

[118] Brzezniak LK, Bijata M, Szczesny RJ, Stepien PP 2011 Involvement of human ELAC2 gene product in 3′ end processing of mitochondrial tRNAs. RNA Biol 8:616–626. doi:10.4161/rna.8.4.15393. 21593607

