# Supplementary material for: Human Cytomegalovirus Infection Upregulates the Mitochondrial Transcription and Translation Machineries
Source: mBio. 2016 Mar 29;7(2):e00029-16. doi: 10.1128/mBio.00029-16 (PMC4807356; doi:10.1128/mBio.00029-16)
Supplement: Text S1 — Supplemental experimental procedures. Download [file mbo002162741s1.docx]

**Supplemental Information**

**Human cytomegalovirus infection upregulates the mitochondrial transcription and translation machineries**

Karniely S, Weekes MP, Antrobus R, Rorbach J, Van Haute L, Umrania Y, Smith DL, Stanton RJ, Minczuk M, Lehner PJ, Sinclair JH

**Supplemental Experimental Procedures**

Reagents:

EtBr was purchased from Amresco, Phosphonoformic acid (PFA), Chloramphenicol and other reagents were purchased from Sigma-Aldrich. The following antibodies were used for Western blot analysis using the indicated dilutions: rabbit anti-28S ribosomal protein S18b (MRPS18b) (Proteintech, 1:2000); mouse anti- 39S ribosomal protein L12 (MRPL12) (Abcam, 1:2000); rabbit anti- RNMTL1 (MRM3)(Sigma-Aldrich, 1:1000); rabbit anti-MRM1 (Sigma-Aldrich, 1;1000); rabbit anti- MTERFD1 (mTERF3) (Sigma-Aldrich, 1:1000); rabbit anti-sera against TFAM was a kind gift from I. Holt (MRC National Institute for Medical Research, London) (1:20,000); rabbit anti VDAC1 (Porin) (Cell Signaling, 1:1000); mouse anti HCMV IE clone [E13] (Argene, 1:6,000) which identifies an epitope shared by HCMV IE1 and IE2, mouse anti-HCMV pp28 (UL99) (Abcam clone [5C3], 1:5000) or (Virusys clone [CH19] , 1:1000); mouse anti- HCMV UL44 (GICR, 1:1,000); rabbit anti-sera against HCMV UL37x1 was a kind gift from A.M. Colberg-Poley (Center for Genetic Medicine Research, Children's National Health System, Washington DC) (DC35, 1:2,500); mouse anti-mt CO1 (Abcam clone [1D6E1A8], 1:1000); mouse anti-mt-CO2 (Life technologies clone [12C4F12], 1:2000); mouse anti-SDHA (Abcam clone [2E3GC12FB2AE2], 1;1000); Total OXPHOS Human WB mouse antibody cocktail (Abcam, 1:1000); rabbit anti-actin (Abcam, 1:1000); rabbit anti-GAPDH (Cell Signaling, 1:2000) .Secondary HRP-conjugated antibodies were obtained from Jackson ImmunoResearch. Secondary Infra-red conjugated antibodies were obtained from (LI-COR Biosciences)

Construction of a Δβ2.7 Merlin HCMV strain

Recombineering was performed as previously described ([1](#_ENREF_1)) using SW102 bacteria. The parental BAC was pAL1111, a BAC containing the complete HCMV strain Merlin genome, with ablating mutations in RL13 and UL128. Recombineering was performed by first PCR amplifying a cassette encoding *amp^r^*, *lacZα* and *sac*B using primers AACAGACCACGAACACGGCAAAAAATGCATGCAAACTTCTCATTTATTGTGTCTACTACTCTGTGTTGCTACAGGGAGCCTGTGACGGAAGATCACTTCG and CACACGTCTTTCCGCTTACTCAACGCGTCAGCCCGCGCTCGGCAGAGCTACCATATAAAAACGCAGGGGTTTAGCGGCTCTGAGGTTCTTATGGCTCTTG, and inserting it into the β2.7 transcript. In a second round of recombineering, the cassette was removed using primer ATGCAAACTTCTCATTTATTGTGTCTACTACTCTGTGTTGCTACAGGGAGAGCCGCTAAACCCCTGCGTTTTTATATGGTAGCTCTGCCGAGCGCGGGCT, deleting the 2,434bp of the β2.7 transcript from the transcriptional start site, to the polyadenylation signal. Virus stocks were generated by electroporation of BAC DNA into fibroblasts using an Amaxa nucleofector (Lonza).

Affinity purification of mitochondria

Affinity purification of mitochondria was based on Hornig-Do et al. ([2](#_ENREF_2)) with some modifications. Following infection the differentially SILAC-labelled cells were washed twice with ice-cold PBS and scraped off in ice-cold PBS and mixed. Cells were pelleted at 300 g and resuspended in 1 ml of ice-cold PEB (PBS, 2 mM EDTA, 0.5% fatty acids free BSA) supplemented with complete protease inhibitor cocktail (Sigma-Aldrich). Cells were homogenized by passing 30 times through a 27 ¾ g needle. The homogenates were spun at 300 g for 5 min at 4°C (without the use of brakes) to clear unbroken cells and nuclei. Supernatants were diluted into 5ml cold PEB and pre-cleared by loading onto a MACS LS Column (Miltenyi Biotec) pre-equilibrated with 3ml PEB. Cleared homogenates were collected, mixed with 50 µl of superparamagnetic microbeads conjugated to anti-TOM22 antibodies (Miltenyi Biotec) and incubated for 60 min at 4°C with slow rotation (magnetic beads were pre- blocked in 500 μl PEB for 60 min at 4°C before addition to homogenates). After incubation homogenates- beads suspensions were loaded onto a fresh MACS LS Column pre-equilibrated with 3ml PEB, which was placed in the magnetic field of a MACS Separator (Miltenyi Biotec). Columns were washed three times with 3 ml of cold PEB buffer+ 0.1M NaCl. After removing the column from the magnetic field, mitochondria were eluted with 1.5ml of PEB buffer +0.1M NaCl by pushing the plunger through the syringe. Mitochondria were then pelleted by centrifugation at 10,000 g for 10 min at 4°C and washed twice with 1 ml of sucrose buffer (0.32 M sucrose, 1 mM EDTA, and 10 mM Tris–HCl, pH 7.5). Mitochondria were solubilized in 50 µl of ST buffer (4% SDS, 100 mM Tris-HCl, pH 7.5) followed by centrifugation at 16,000 g to remove microbeads. Supernatants were collected and kept at -80°C until processing for MS analysis. Of note, lysis buffer did not contain any reducing agent to avoid release of the anti-tom22 light chains which may have heavily contaminated the samples.

In our hands the use of affinity purification of mitochondria was superior to both crude differential centrifugation and separation using a two-step sucrose gradient in terms of purity and yield of mitochondria (our unpublished results).

Additional SILAC Data Analysis

To estimate any potential bias introduced by different total amounts of heavy and light-labelled mitochondrial proteins. We employed two complementary strategies:

Strategy 1: Remove all non-mitochondrial proteins from our existing data, keeping all mitochondrial proteins (as annotated in Mitocarta). Next, sum the intensities of all heavy, and all light-labelled mitochondrial proteins, and calculate a correction factor to account for differences in the summed abundance of mitochondrial proteins in each sample. Correction factors were small (1.40 for experiment A (Table S1A) and 1.20 for experiment B (Table S1B).

Strategy 2:Re-search all raw mass spec files using MaxQuant, employing 3 databases: a. All human proteins, b. All human proteins with mitochondrial proteins removed and c. mitochondrial proteins (annotated in Mitocarta). We included database (b) since searching data against all mitochondrial proteins alone (c) might increase the false discovery rate, as many searched peptides would no longer have a database match. We saw very good correlation between pairwise comparison of ratios, for data searched against (b) vs (a) and (c) vs (a) (Figure S3B). By comparing the latter searches, ratio correction factors equivalent to those shown in Strategy 1 above could be calculated from (1/gradient), and were 1.06 (experiment A) and 1.20 (experiment B). Correction factors were smaller when comparing searches against databases (b) and (a) since potential mitochondrial peptides only account for a small proportion of all human peptides.

siRNA knockdown of MRM3

TriFECTa RNAi duplexes were obtained from Integrated DNA Technologies (IDT). MRM3 RNAi duplexes were composed of following sequences: NM_018146 MRM3 duplex 1 Sense rArCrCrArUrGrUrUrArArGrArUrGrArCrArUrArUrCrCrAAA and NM_018146 MRM3 duplex 1 Antisense rUrUrUrGrGrArUrArUrGrUrCrArUrCrUrUrArArCrArUrGrGrUrCrA. We have also tested the following custom made duplexes based on ([3](#_ENREF_3)) MRM3 Custom Sense ArGrGrCrUrCrArUrUrUrCrArGrArCrGrCrUrCrUrCrArAGG and MRM3 Custom Antisense rCrCrUrUrGrArGrArGrCrGrUrCrUrGrArArArUrGrArGrCrCrUrGrC this duplex allowed partial knockdown of MRM3 at 48hpi and none at 5dpi (our unpublished data) and therefore was not used in the experiment described in Fig. 6.

Total cell extraction for immunoblotting

Cells were washed twice with ice cold PBS and lysed with cold RIPA buffer (1% NP-40, 1% Sodium Deoxycholate, 50 mM NaCl, 25 mM Tris-HCl, pH 8, 0.1% SDS) or IP buffer (1% Tx-100, 1% Sodium Deoxycholate, 50 mM NaCl, 25 mm Tris-HCl, pH 8) supplemented with complete protease inhibitor cocktail (Sigma-Aldrich) for 5 min at 4°C followed by 2 cycles of sonication. Cell lysates were spun at 14,000g for 10 min at 4°C and cleared supernatants were collected. Protein concentration was determined using a BCA Protein assay kit (Pirece) and samples were normalized for their protein content with lysis buffer before loading on polyacrylamide gels.

Gel electrophoresis and Western blotting

Samples were supplemented with Laemmli loading buffer (containing DTT) and proteins were separated by electrophoresis using 10% or 12% polyacrylamide gels or 4-12% Bis-Tris Plus gels (Life technologies). Samples were electro-transferred to nitrocellulose or PVDF membranes in Towbin transfer buffer (25 mM Tris-HCl, pH 8.3, 192 mM glycine, 20% (v/v) methanol). Membranes were blocked with 5% milk in TBST (10 mM Tris-HCl, pH 7.4, 150 mM NaCl, and 0.3% Tween 20) rinsed with TBST and sequentially incubated with the primary antibodies (2 h at RT or 16 h at 4°C) and the secondary antibodies (45 min at RT), both in 5% BSA in TBS. Membranes were then rinsed extensively in TBST, developed with ECL reagents and imaged, except for Fig. S6D where fluorescent secondary antibodies were used and detected with Odyssey CLx (LI-COR Biosciences). In some experiments nitrocellulose membranes were stripped from both primary and secondary antibodies using Restore Plus Western Blot Stripping Buffer (Pirece) for 15 min at 37 °C and then washed with PBS, blocked, and re-probed with other antibodies. Densitometric analysis to quantify protein bands on Western blots was performed using ImageJ (NIH, Bethesda, Maryland) except for Fig. S6D where Image Studio (LI-COR Biosciences) was used.

Mitochondrial translation analysis after emetine pre-treatment

In the experiment shown in Fig. S6C, cells were treated with chloramphenicol immediately after virus inoculation and until radiolabeling to increase mt-labelling. Four hours before labelling cells were either pre-treated with emetine or left untreated followed by radiolabeling (without chloramphenicol) as described in Material and Methods. Cells were lysed with 0.1% DDM, 50 units of Benzonase supplement with complete protease inhibitor cocktail (Sigma-Aldrich) and SDS was added to 1%. Lysates were separated on a 15% SDS–PAGE after which the gel was stained with Coomassie Brilliant Blue G-250 (to confirm equal loading), dried, exposed to a Phosphorimager screen and visualizes using a FLA5100 scanner (Fujiimager, Tilburg, The Netherlands).

RNA extraction and Quantitation on MRM3 and TFB2M mRNA levels

Total RNA was extracted from cells using RNeasy Mini Kit (Qiagen) according to the manufacturer’s instructions. RNA was converted to cDNA using a High capacity cDNA reverse transcription kit (Applied biosystems) and relative cDNA amounts were measured by qPCR using the 2^-ΔΔCt^ method using GAPDH and POLR2L as reference genes.

DNA extraction and quantitation on mt-DNA

Total (Cellular and mitochondrial) DNA was extracted from cells as described previously ([4](#_ENREF_4)). Briefly, cells were resuspended in PCR Solution A (100 mM KCl, 10 mM Tris-HCl [pH 8.3], 2.5 mM MgCl_2_). Cells were pelleted and then washed with PCR Solution A. After pelleting, cells were resuspended in a 1:1 mixture of PCR Solution A and PCR Solution B (10 mM Tris-HCl [pH 8.3], 2.5 mM MgCl2; 1% Tween-20; 1% Nonidet P-40; 0.4 mg/mL proteinase K). The samples were incubated at 60^o^C for 60 minutes with intermittent vigorous agitation, followed by incubation at 100^o^C for 10 minutes to inactivate proteinase K. Organic extractions and ethanol precipitations were not required for downstream PCR analysis. Relative copy numbers of mt-DNA were determined by qPCR using the 2^-ΔΔCt^ method.

qPCR

For quantitation of MRM3 and TFB2M cDNA an input of 25 ng cDNA was used with 2x Fast SYBR Green Master Mix (Applied Biosystem) and 250 nM of forward and reverse primers. Samples were run on a StepOnePlus real time PCR machine (Applied Biosystems) using the following qPCR protocol: Initial 20 sec at 95°C followed by 40 cycles of 95°C for 3 sec and 30 sec at 60°C. Samples were measured in triplicates. The following primer sets were used: MRM3 For AGGGTCACGGAGATTGTCAC and MRM3 Rev AACGCCACAAGGAATAATGG, TFB2M For TCACTCGTAGTTTTCCATCC and TFB2M Rev ACTTGAAGCTGGTGCCAAAG, GAPDH For AATGAAGGGGTCATTGATGG and GAPDH Rev AAGGTGAAGGTCGGAGTC, POLR2L For AGGAGAGCCTTCCATCTCG and POLR2L Rev ATCTGGCTCTTCAGATTCCG. Fold increase over mock infected cells was calculated using 2^-ΔΔCt^ method. GAPDH and POLR2L were used as reference genes.

For quantitation of mt-DNA an input of 100 ng DNA was used with 2X TaqMan Gene Expression Master Mix (Life Technologies), 900 nM forward and reverse primers and 250 nM probe (Sigma Aldrich) and nuclease-free water up to a total volume of 25 µL. Samples were run in a ABI 7900HT cycler using the following qPCR protocol: Initial 2 min at 50°C and 10 min at 95°C followed by 40 cycles of 95°C for 15 sec and 1 min at 60°C. Samples were measured in triplicates. Fold increase over mock infected cells was calculated using 2^-ΔΔCt^ method. All probes were 6-FAM/TAMRA labelled. For mt-DNA we used two sets of primers and probes. Set 1: mt3211f CACCCAAGAACAGGGTTTGT; mt3298r TGGCCATGGGTATGTTGTTAA; mt3242 probe TTACCGGGCTCTGCCATCT. Set 2: mt9827f CGTCATTATTGGCTCAAC; mt9974r GATGGAGACATACAGAAATAG; mt9852 probe ACTATCTGCTTCATCCGCCACTAA. Nuclear genomic B2M was used as reference using the following set: B2Mf TGCTGTCTCCATGTTTGATGTATCT, B2Mr; TCTCTGCTCCCCACCTCTAAGT and B2Mprobe TTGCTCCACAGGTAGCTCTAGGAGG.

Virus titration

Tissue culture infectious dose 50 (TCID_50_) was used to determine virus titres in media collected from infected cells. Briefly, samples were serially diluted (log dilutions) in medium in a 96 well plate each sample at six replicates. 100 μl of each dilution was then added onto 10,000 HFFF2 cells pre-seeded a day before in a 96 well plate. The plates were incubated for 10 days and the dilution at which point no plaques could be observed was determined for each serial dilution. TCID_50_ was calculated using the Reed and Muench method ([5](#_ENREF_5)). To convert TCID_50_ into pfu/ml a conversion factor of 0.7 was applied. Statistical analysis was performed using Prism 5.0 (GraphPad Software, Inc, San Diego, CA).

**Supplemental References**

1. **Stanton RJ, Baluchova K, Dargan DJ, Cunningham C, Sheehy O, Seirafian S, McSharry BP, Neale ML, Davies JA, Tomasec P, Davison AJ, Wilkinson GW.** 2010. Reconstruction of the complete human cytomegalovirus genome in a BAC reveals RL13 to be a potent inhibitor of replication. J Clin Invest **120:**3191-3208.

2. **Hornig-Do HT, Gunther G, Bust M, Lehnartz P, Bosio A, Wiesner RJ.** 2009. Isolation of functional pure mitochondria by superparamagnetic microbeads. Anal Biochem **389:**1-5.

3. **Rorbach J, Boesch P, Gammage PA, Nicholls TJ, Pearce SF, Patel D, Hauser A, Perocchi F, Minczuk M.** 2014. MRM2 and MRM3 are involved in biogenesis of the large subunit of the mitochondrial ribosome. Mol Biol Cell **25:**2542-2555.

4. **Roback JD, Hillyer CD, Drew WL, Laycock ME, Luka J, Mocarski ES, Slobedman B, Smith JW, Soderberg-Naucler C, Todd DS, Woxenius S, Busch MP.** 2001. Multicenter evaluation of PCR methods for detecting CMV DNA in blood donors. Transfusion **41:**1249-1257.

5. **Reed LJ, Muench H.** 1938. A simple method of estimating fifty per cent endpoints. American Journal of Epidemiology **27:**493-497.
