# Supplementary figures and images for: Human Cytomegalovirus Infection Upregulates the Mitochondrial Transcription and Translation Machineries
Source: mBio. 2016 Mar 29;7(2):e00029-16. doi: 10.1128/mBio.00029-16 (PMC4807356; doi:10.1128/mBio.00029-16)

Figure S1

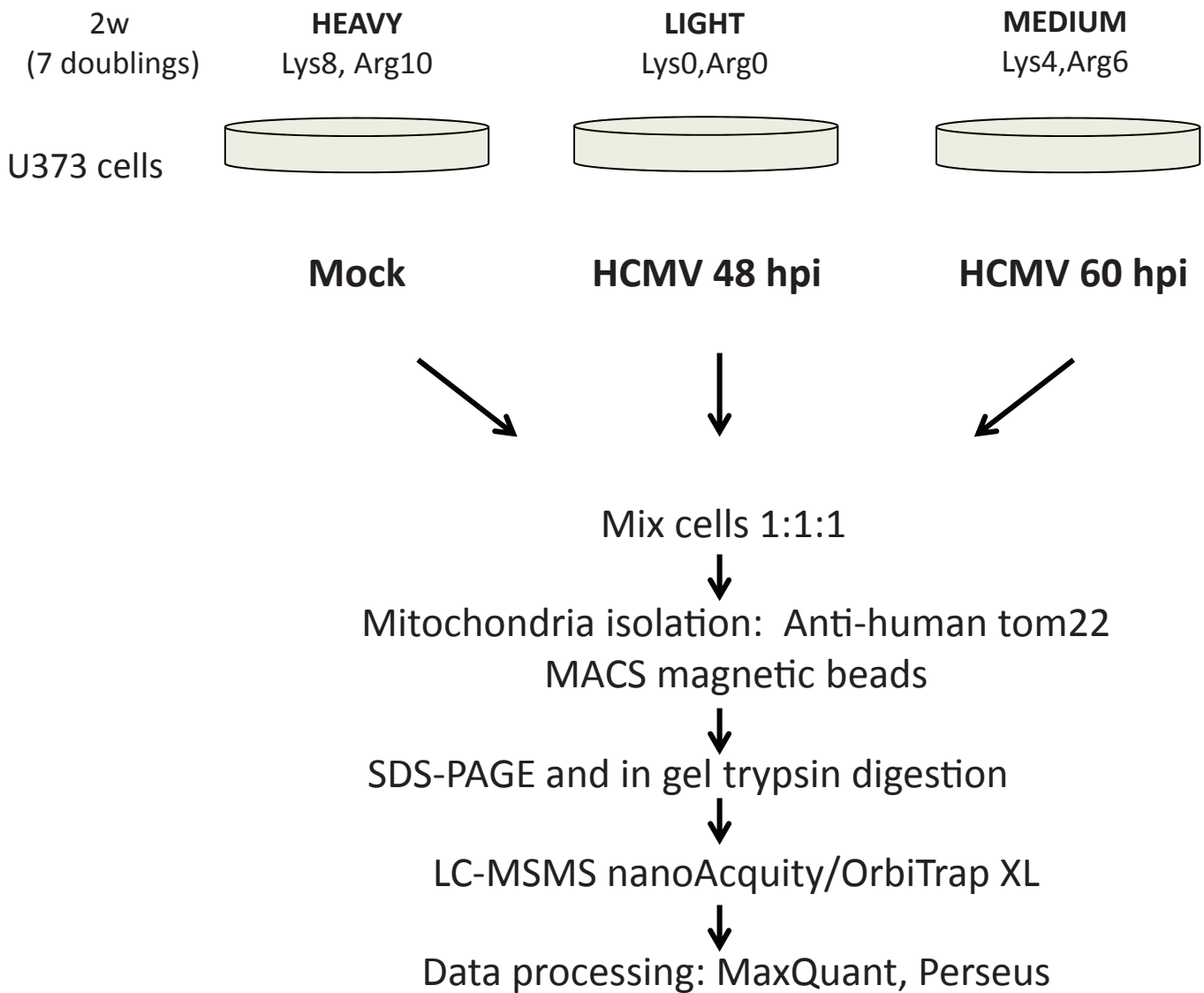

Supplement: Figure S1 — Work flow of SILAC-MS analysis of the mitochondrial proteome following HCMV infection. U373 cells were cultured for 7 passages in “light,” “heavy,” or “medium” SILAC-DMEM. Cells were infected at an MOI of 3 with HCMV strain Merlin. The 48-h and 60-h infections were staggered such that all flasks were harvested simultaneously. Prior to mitochondrial isolation, the 3 cell populations were mixed. Cells were then homogenized, and mitochondria were isolated by affinity purification using superparamagnetic microbeads conjugated to anti-TOM22 antibodies. Purified mitochondria were lysed, and extracted proteins were separated by SDS-PAGE and trypsin digested. Peptides were eluted and analyzed by LC-MS/MS Download [file mbo002162741sf1.pdf]

Figure S2

A

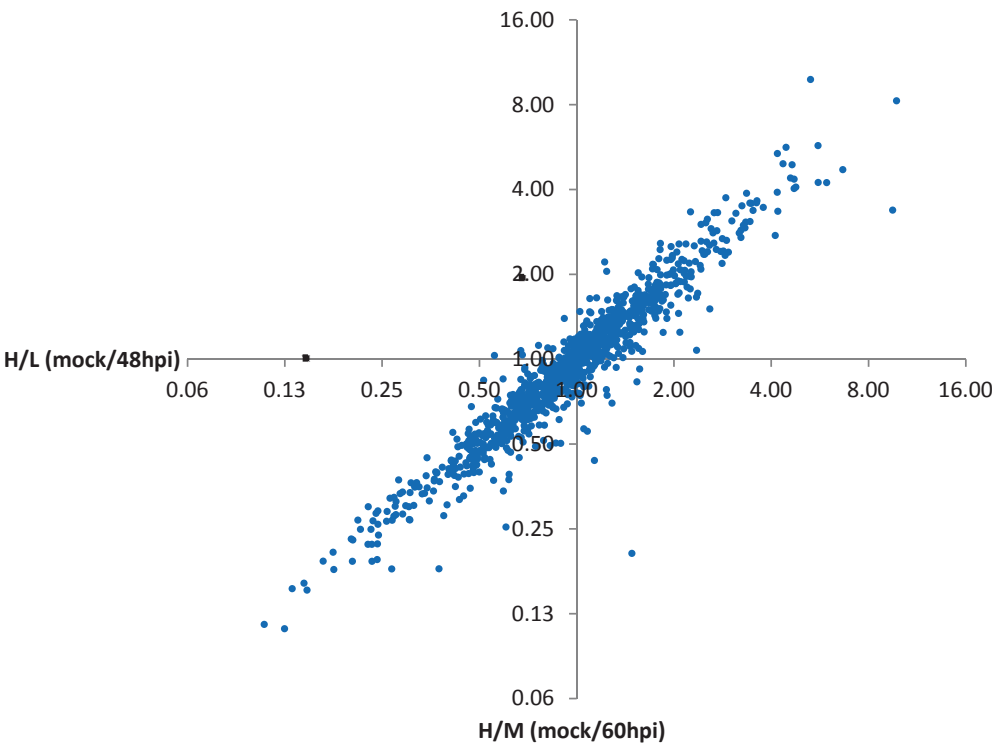

B

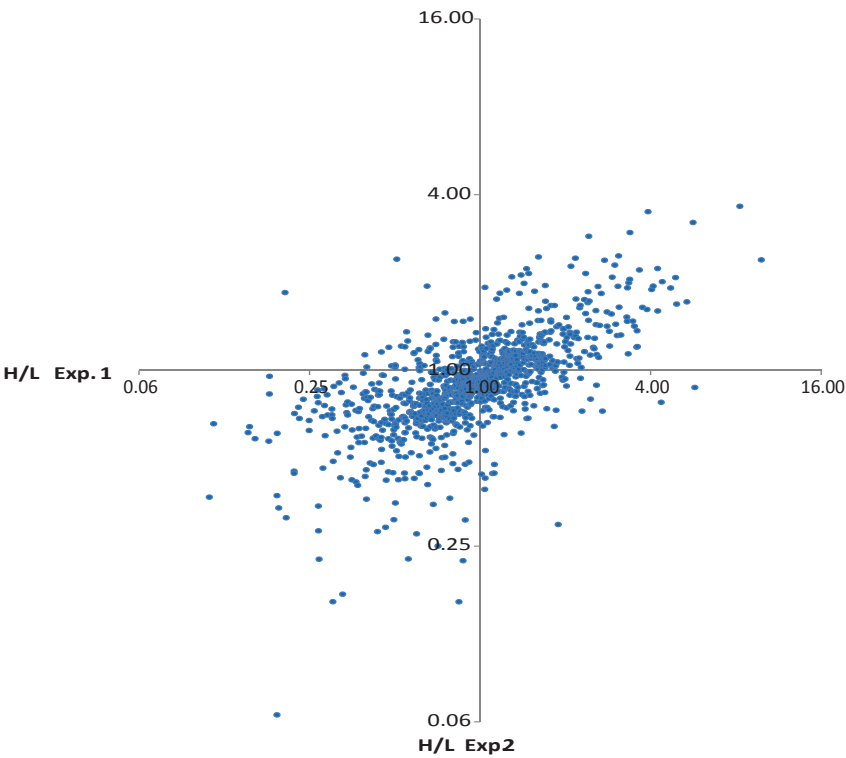

C

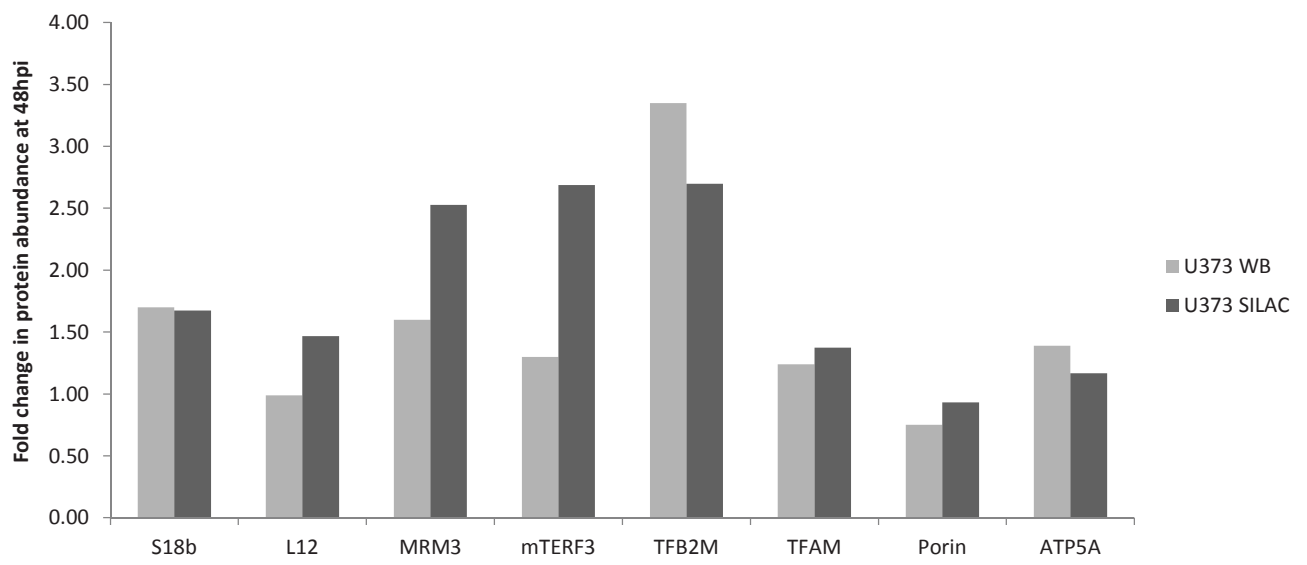

Supplement: Figure S2 — (A) Comparison of the mitochondrial proteome of U373 cells between 48 and 60 h of HCMV infection. Shown is a scatter plot representing the fold change in “heavy” (mock)/“medium” (HCMV 60 hpi) (y axis) compared to “heavy” (mock)/“light” (HCMV 48 hpi) (x axis), with both axes displayed using a log2 scale. The changes in abundance of mitochondrially associated proteins relative to those in mock-infected cells were nearly identical after 48 and 60 h of HCMV infection. (B) An independent biological repeat of the SILAC experiment shows similar changes in the mitochondrial proteome following HCMV infection. Shown is a scatter plot representing the fold change in “heavy” (mock)/“light” (HCMV 48 hpi) in two independent biological repeats, with both axes displayed using a log2 scale. Protein ratios show a high degree of correlation between experiments. (C) WB measurements correlate with SILAC data. The fold changes in abundance of mitochondrial proteins at 48 hpi presented in Fig. 2 were assessed by densitometric analysis and compared to the values obtained in the SILAC-MS analysis (average of values from Table S1A and S1B). Download [file mbo002162741sf2.pdf]

Figure S3

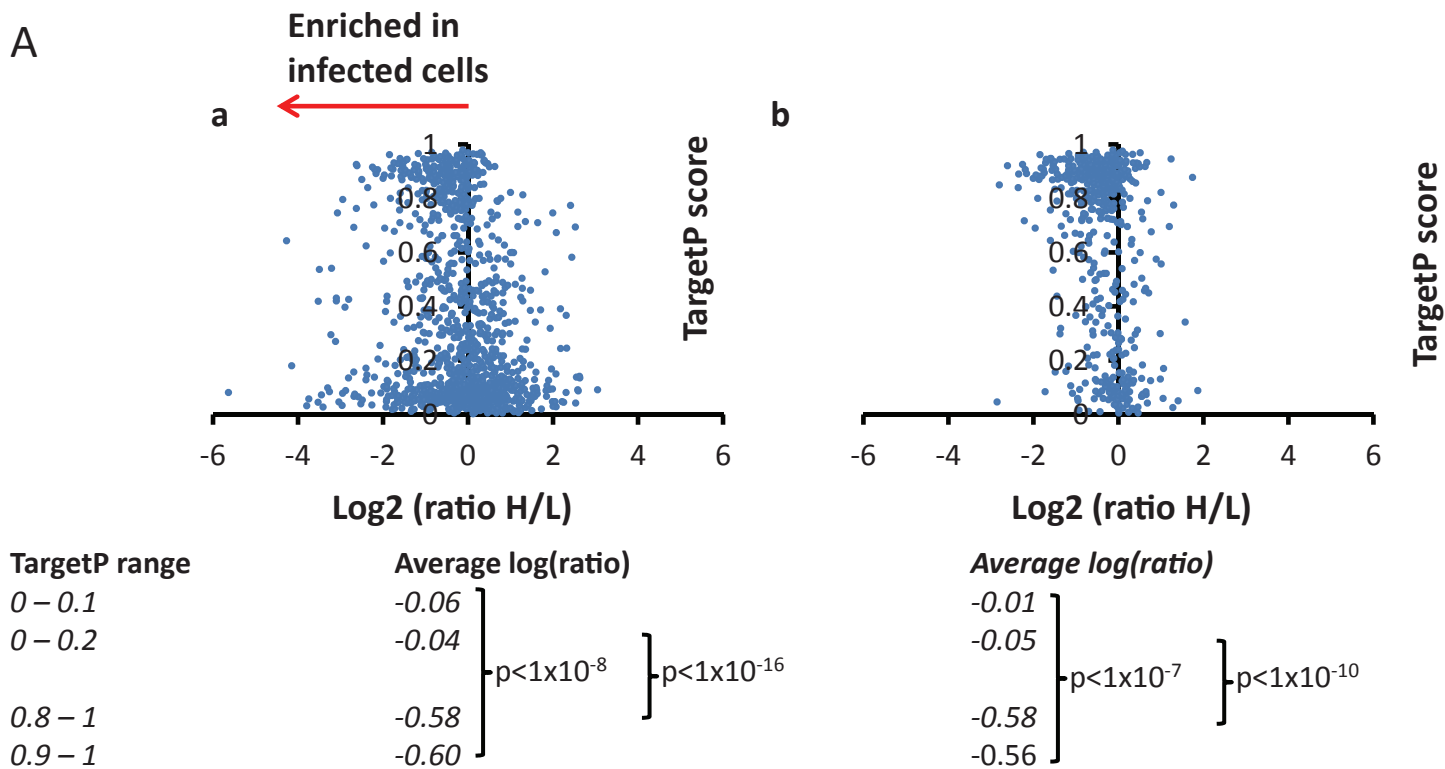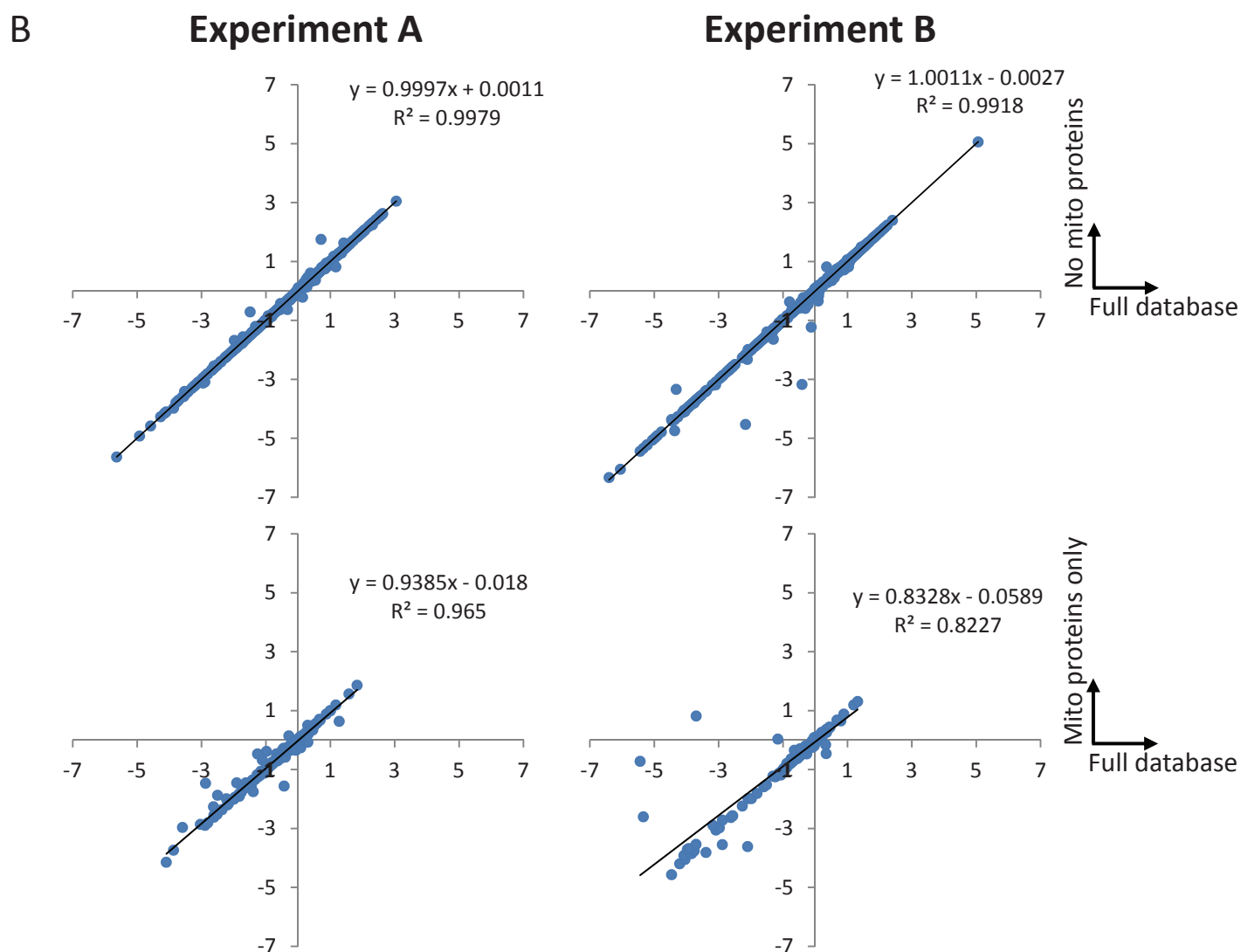

Supplement: Figure S3 — (A) Proteins with high mTP scores are enriched in infected cells. TargetP scores were plotted against log (H/L ratio) for the whole UniProt database (a) and against the UniProt database of mitochondrial proteins only (annotated by Mitocarta [b]). Proteins with a negative log ratio were relatively enriched in infected cells, which were labeled with light amino acids (red arrow). The significance of difference in the average log (ratio) between populations defined by TargetP 0 to 0.1 and 0.9 to 1 or TargetP 0 to 0.2 and 0.8 to 1 was calculated using a 2-tailed t test (2 samples, unequal variance). (B) Fold change measurements were not biased by differences in total amounts of heavy- and light-labeled mitochondrial proteins. Raw MS files were searched using MaxQuant, and fold changes in protein abundance at 48 hpi were calculated, employing 3 databases: a, all human proteins; b, all human proteins with mitochondrial proteins removed; c, all mitochondrial proteins only (annotated by Mitocarta). Pairwise comparison of ratios for data searched against databases b versus a and c versus a are presented. Download [file mbo002162741sf3.pdf]

Figure S4

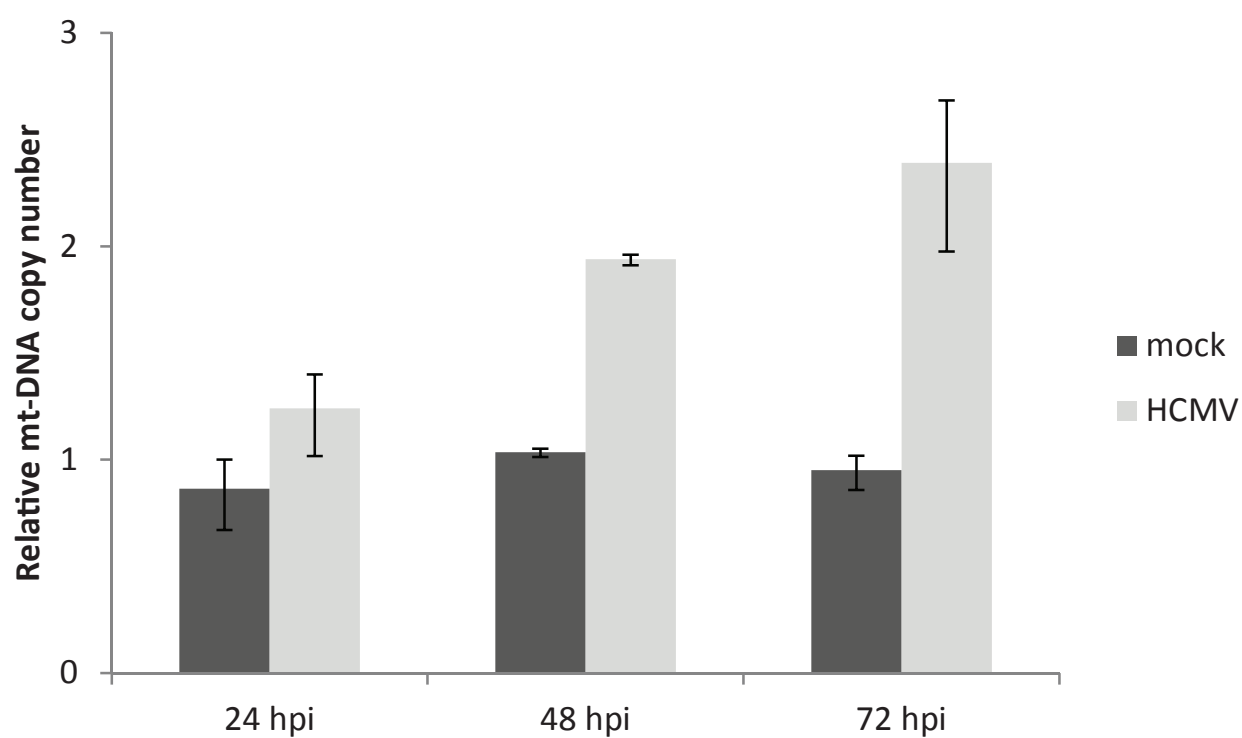

Supplement: Figure S4 — mtDNA is upregulated at late stages of HCMV replication. HFFF2 cells were either mock infected or infected with HCMV at an MOI of 5 for 24, 48, or 72 h. Total DNA was extracted from cells, and the relative expression of mtDNA was determined using quantitative PCR (qPCR). Column charts show the fold change in mtDNA compared to that in mock-infected cells at 24 hpi. Error bars represent standard errors from two biological repeats. Download [file mbo002162741sf4.pdf]

Figure S5

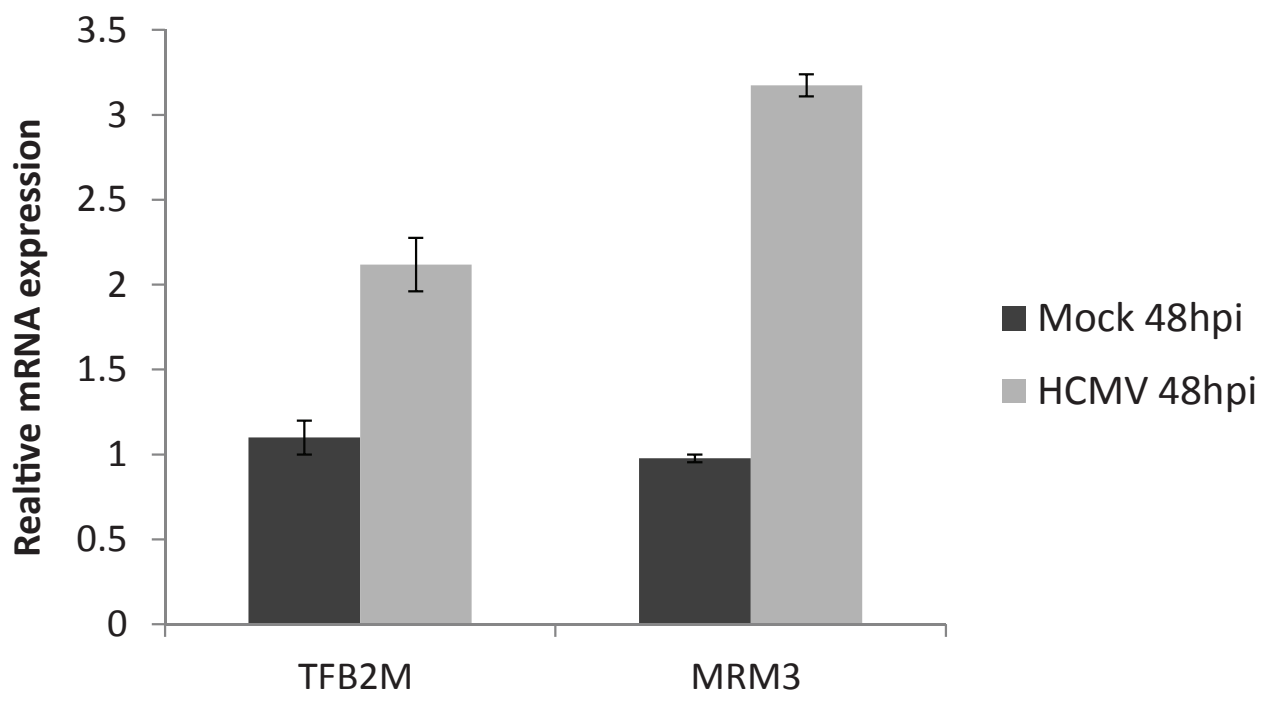

Supplement: Figure S5 — TFB2M and MRM3 are induced at the level of mRNA following HCMV infection. HFFF2 cells were either mock infected or infected with HCMV at an MOI of 5 for 48 h. Total RNA was extracted from cells, and the relative expression of TFB2M and MRM3 mRNAs was determined using reverse transcription-quantitative PCR (RT-qPCR) using GAPDH and POLR2L as the reference genes. Column charts show the fold change in mRNA expression in HCMV-infected over mock-infected cells. Error bars represent standard errors from two biological repeats. Download [file mbo002162741sf5.pdf]

Figure S6

A

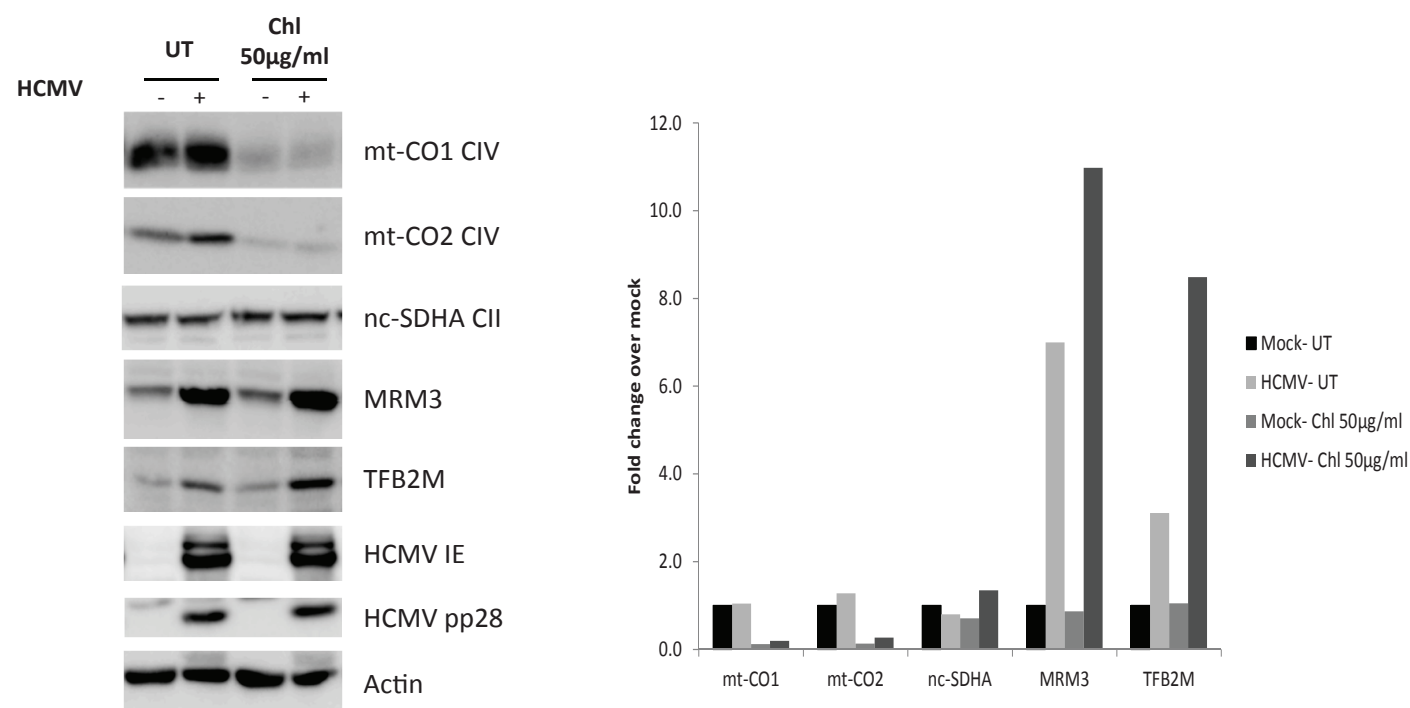

B

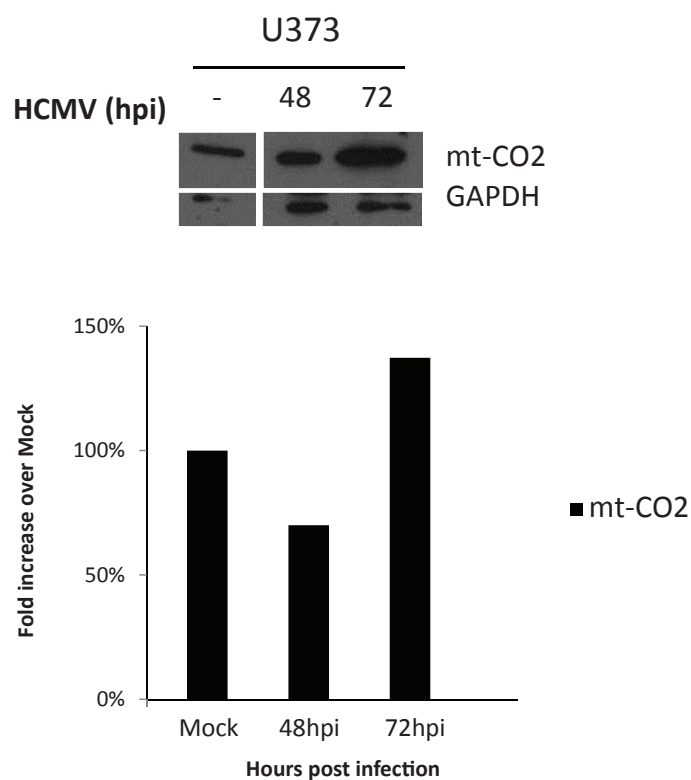

C

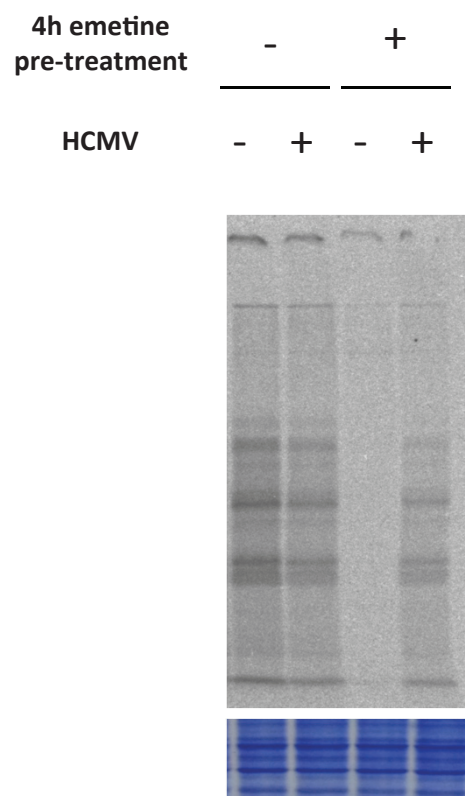

D

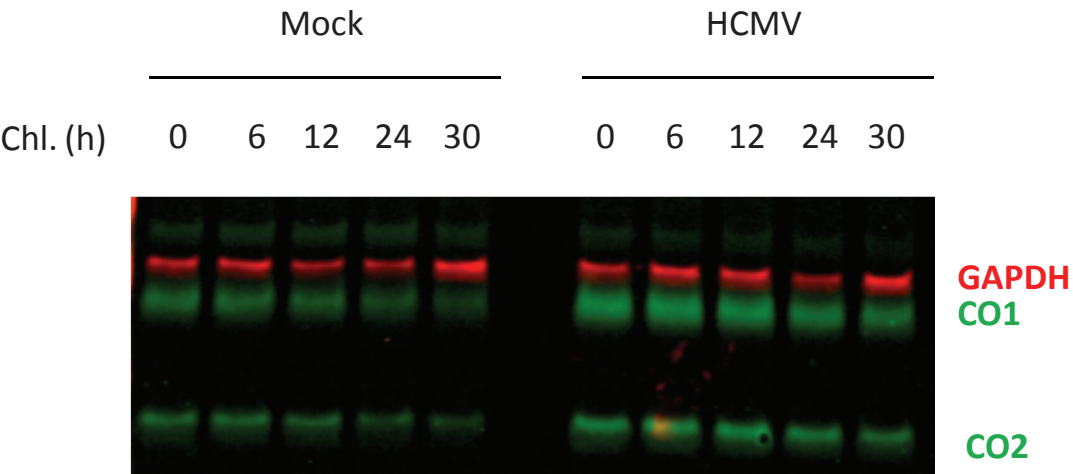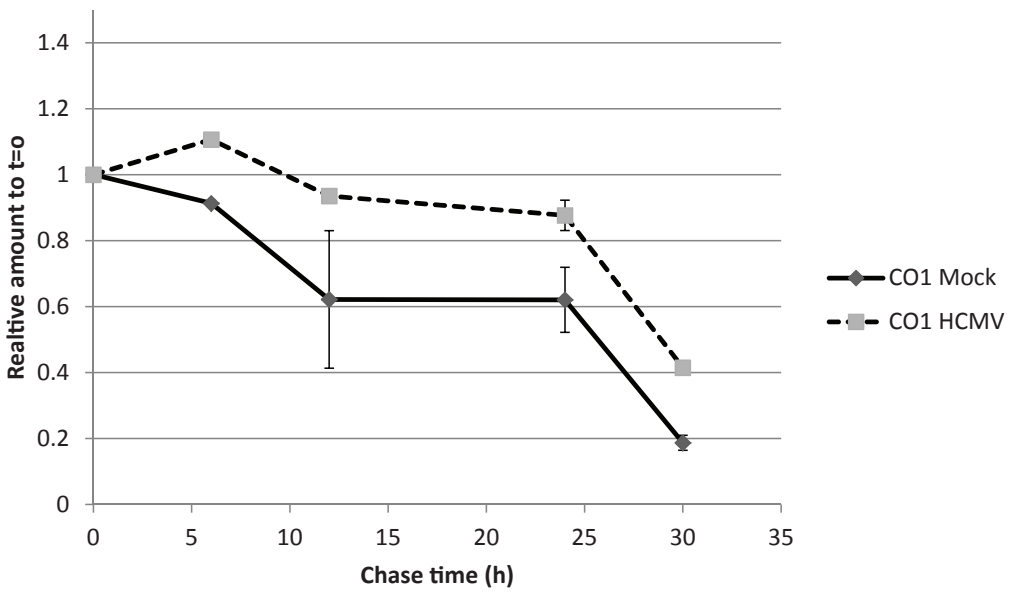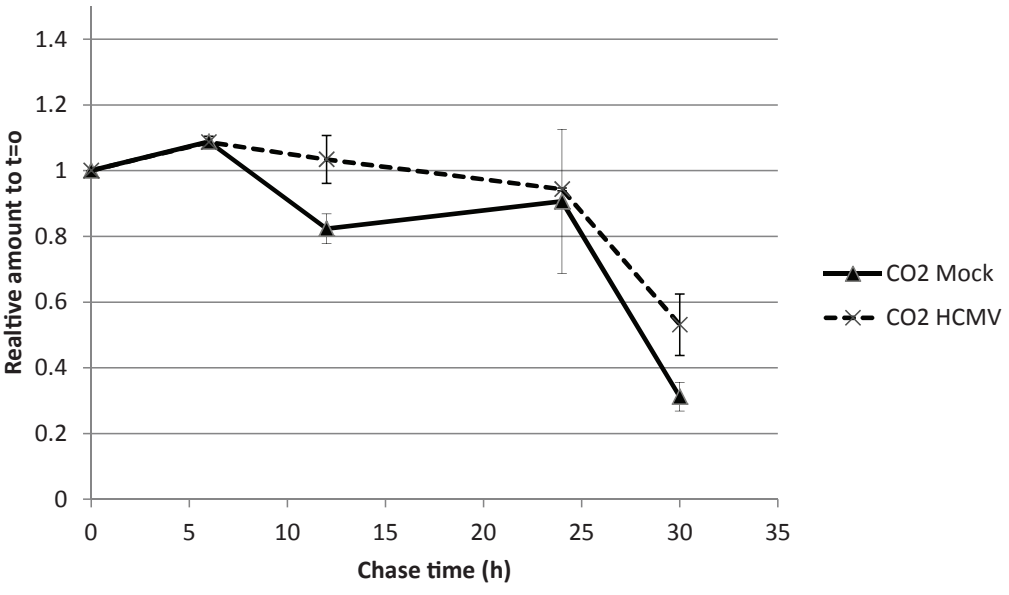

E

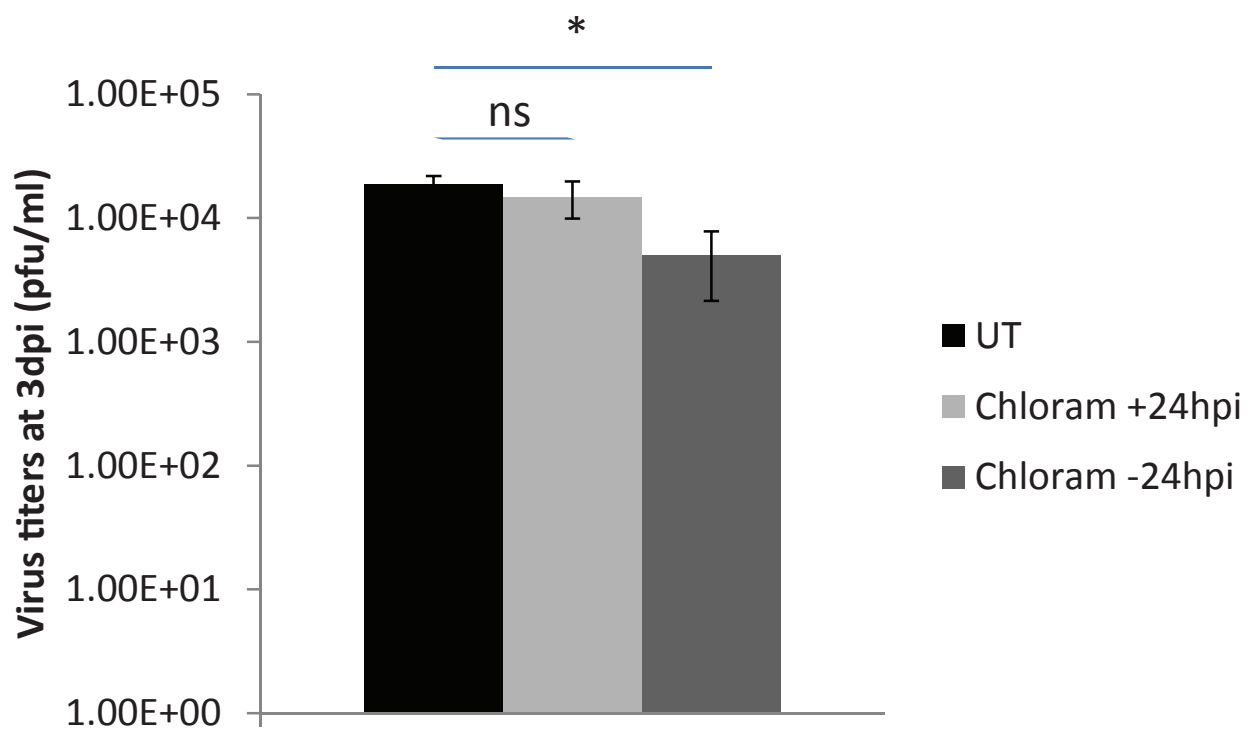

Supplement: Figure S6 — (A) Induction of mitochondrially encoded proteins by HCMV was blocked by chloramphenicol. HFFF2 cells were either mock infected or infected with HCMV at an MOI of 5. After 2 h, the inoculum was washed and cells were refreshed with untreated medium (UT) or with medium containing chloramphenicol to block mitochondrial translation. Cells were harvested at 48 hpi, and lysates were processed and analyzed by WB. (B) mt-CO2 was induced in infected U373 cells only at 72 hpi. HFFF2 cells were either mock infected or infected with HCMV for 48 or 72 h. Cells were harvested and lysates were processed and analyzed by WB. The charts in panels A and B show the fold changes in protein abundance after viral infection. (C) Blocking cytosolic translation abrogated mitochondrial translation in mock-infected but not in HCMV-infected cells. Mock-infected and HCMV-infected cells were radiolabeled with [35S]methionine either with or without 4 h of pretreatment with emetine (which blocks cytosolic translation). Total cell lysates were separated by 15% SDS-PAGE. Equal loading of the gels was confirmed by staining the gel with Coomassie brilliant blue G-250. Dried gels were exposed to phosphorimager plates and scanned. (D) Mitochondrially encoded proteins are stabilized by HCMV infection. Mock-infected or HCMV-infected HFFF2 cells were treated with chloramphenicol at 24 hpi for the times indicated. Cell lysates were analyzed by WB. Graphs show the fold change in mt-CO1 and mt-CO2 over time relative to t = 0 (addition of chloramphenicol) normalized to GAPDH. (E) Pretreatment with chloramphenicol reduces viral growth on glucose-fed cells. Mock-infected or HCMV-infected HFFF2 cells were grown in DMEM supplemented with 5 mM glucose (as described in the legend to Fig. 6A) and were either left untreated or treated with chloramphenicol (50 µg/ml) either 24 h prior to or 24 h after HCMV infection. At 3 dpi, media were collected from cells, and released virus titers were quantified using TCID50. Error [file mbo002162741sf6.pdf]
